# Silencing S-Adenosyl-L-Methionine Decarboxylase (SAMDC) in *Nicotiana tabacum* Points at a Polyamine-Dependent Trade-Off between Growth and Tolerance Responses

**DOI:** 10.3389/fpls.2016.00379

**Published:** 2016-03-31

**Authors:** Ifigeneia Mellidou, Panagiotis N. Moschou, Nikolaos E. Ioannidis, Chryssa Pankou, Katalin Gėmes, Chryssanthi Valassakis, Efthimios A. Andronis, Despoina Beris, Kosmas Haralampidis, Andreas Roussis, Aikaterini Karamanoli, Theodora Matsi, Kiriakos Kotzabasis, Helen-Isis Constantinidou, Kalliopi A. Roubelakis-Angelakis

**Affiliations:** ^1^Department of Crop Production, School of Agriculture, Aristotle University of ThessalonikiThessaloniki, Greece; ^2^Department of Plant Biology and Linnean Center of Plant Sciences, Uppsala BioCentrum, Swedish University of Agricultural SciencesUppsala, Sweden; ^3^Department of Biology, University of CreteHeraklion, Greece; ^4^Biological Research Centre, Hungarian Academy of SciencesSzeged, Hungary; ^5^Department of Biology, National and Kapodistrian University of AthensAthens, Greece

**Keywords:** polyamine metabolism, salt tolerance, silenced SAMDC, SAMDC transgenics, ROS, transgenic assessment

## Abstract

Polyamines (PAs) are nitrogenous molecules that are indispensable for cell viability and with an agreed-on role in the modulation of stress responses. Tobacco plants with downregulated SAMDC (AS-*SAMDC*) exhibit reduced PAs synthesis but normal levels of PA catabolism. We used AS-*SAMDC* to increase our understanding on the role of PAs in stress responses. Surprisingly, at control conditions AS-*SAMDC* plants showed increased biomass and altered developmental characteristics, such as increased height and leaf number. On the contrary, during salt stress AS-*SAMDC* plants showed reduced vigor when compared to the WT. During salt stress, the AS-*SAMDC* plants although showing compensatory readjustments of the antioxidant machinery and of photosynthetic apparatus, they failed to sustain their vigor. AS-*SAMDC* sensitivity was accompanied by inability to effectively control H_2_O_2_ levels and concentrations of monovalent and divalent cations. In accordance with these findings, we suggest that PAs may regulate the trade-off between growth and tolerance responses.

## Introduction

Polyamines (PAs) exert pleiotropic functions that can be best seen during stress responses. They are implicated in a multitude of processes including protein translation, DNA packing, redox state regulation, photosynthesis, and nitrogen content balancing. Regulation of PA turnover involves PA biosynthesis, oxidation/back-conversion, conjugation, transport, and compartmentalization (Minocha et al., 2014 and references therein).

**Figure 1:**
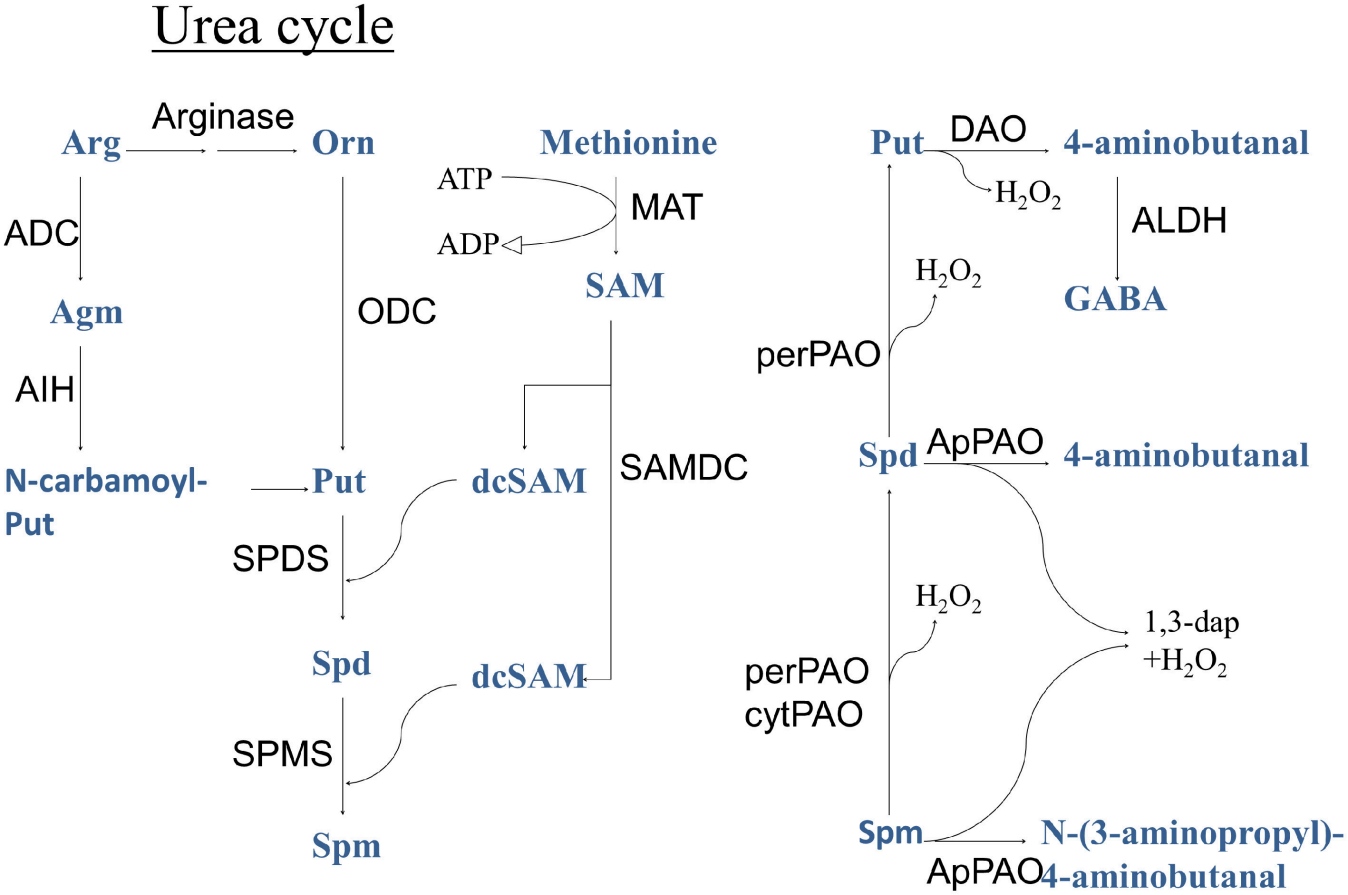
**Polyamine metabolic pathway**. ADC, arginine decarboxylase; ALDH, aldehyde dehydrogenase; Cad, cadaverine; CuAO, copper amine oxidase; dcSAM, decarboxylated S-adenosyl-L-methione; LDC, lysine decarboxylase; MAT, methionine adenosyltransferase; ODC, ornithine decarboxylase; PAO, polyamine oxidase (per, peroxisomal; ap, apoplastic; cyt, cytoplasmic); SAM, S-adenosyl-L-methione; Spd, spermidine; Spm, spermine; PMT, putrescine *N*-methyl transferase; Put, putrescine; SAMDC, S-adenosyl-L-methione decarboxylase; SPDS, spermidine synthase; SPMS, spermine synthase.

During PA biosynthesis, S-adenosyl-L-methionine decarboxylase (SAMDC, EC 4.1.1.50) converts the methyl donor and ethylene precursor S-adenosyl-L-methionine (SAM), to decarboxylated S-adenosyl-L-methionine (dcSAM; Figure 1). Decarboxylated SAM serves as an aminopropyl donor in the synthesis of higher PAs, the triamine Spermidine (Spd), and the tetramine Spermine (Spm) from the diamine Putrescine (Put). These PAs are catabolized by apoplastic PA oxidases (PAOs) producing the reactive oxygen species (ROS), hydrogen peroxide (H_2_O_2_). In addition, peroxisomal and cytoplasmic PAOs are involved in the conversion of higher PAs to Put (Moschou et al., 2008c,d).

Deregulation of *SAMDC* activity affects plant development. For example, in potato downregulation of *SAMDC* results to stunted phenotype with branched stems, short internodes, small leaves and inhibited root growth (Kumar et al., 1996). These phenotypes are associated with decreased PA content and increased ethylene evolution. In *Arabidopsis thaliana* downregulation of *SAMDC* resulted in decreased higher PAs content but increased Put; these changes were not associated with observable phenotypes (Hu et al., 2006). Finally, rice transgenics downregulating *SAMDC* exhibited reduced Spd and Spm and PAO activity, and reduced apical dominance, decreased plant height, higher tiller numbers, reduced fecundity and tolerance to abiotic stresses (Chen et al., 2014).

Previously, we showed that *Nicotiana tabacum* (tobacco) seedlings with downregulated *SAMDC* (AS-*SAMDC*) show reduced tolerance to salt stress (NaCl), associated with a decrease in the ratio between biosynthesis/catabolism of PAs (Moschou et al., 2008b). In an attempt to increase our understanding on how the ratio of biosynthesis/catabolism of PAs can affect stress tolerance, we present here a detailed characterization of AS-*SAMDC* plants during NaCl stress performed in different plant growth stages at both, the molecular/biochemical and the whole plant level. Our results further support the protective role of PAs in stress tolerance, and suggest that photosynthesis and antioxidant machinery are the main targets of PAs during stress. In addition, we show that PAs may regulate a trade-off between biomass production and stress tolerance, at least in some species.

## Materials and methods

### Plant material and growth conditions

Preparation of AS-*SAMDC* plants (*N. tabacum* cv Xanthi) was described previously (Moschou et al., 2008b). In order to achieve synchronous germination, seeds of both genotypes, WT and AS-*SAMDC* were stratified (4°C, 7 days), and immersed in KNO_3_ (1%) solution for 12 h. Seeds were surface sterilized with bleach for 10 min, washed four times with sterile water and plated out on a filter paper in plates containing half strength Murashige and Skoog medium [Duchefa Biochemie, Haarlem, Netherlands; supplemented with 0.05% MES/KOH, pH 5.7 (2-(*N*-morpholino) ethanesulfonic acid; Sigma-Aldrich, St. Louis MO, USA), Gamborg B5 vitamins and micronutrient mixture (Duchefa), 2% (w/v) sucrose]. Seeds were germinated and grown under long day conditions [16/8 h photoperiod; 22/18°C, 110 μEm^−2^s^−1^ PAR supplied by cool-white fluorescent tungsten tubes (Osram, Berlin, Germany)] in a plant growth chamber for 13 days. Seedlings were transplanted in plates with the same medium containing 0 or 100 mM NaCl for 2 more days. The seedlings were used immediately or weighed, frozen in liquid nitrogen and stored at −80°C for further use.

Responses at the whole plant level were studied using potted plants, which received NaCl treatments for 21 days. Stratified seeds, were sown in individual 10-cm diameter pots filled with peat (Terraplant, Compo) and perlite (2:1) mixture, and placed under plastic cover for the first 3 days to sustain high relative humidity. At the four-true-leaf stage, 120 seedlings per genotype were transplanted in 0.25 L pots, filled with the aforementioned mixture and placed in a completely randomized design (split-split-plot arrangement). At the 4–5 pair true leaf stage (70 days-old plants), five salt treatments (0, 50, 100, 200, 300 mM NaCl) were applied (24 plants per genotype). All plants were subjected to salt treatment through irrigation either with 100 mL aqueous solution of the respective NaCl concentration or with 100 mL water (controls) at 0, 4, 7, 11, 14, and 18 days (days after treatment, DAT). Preliminary experiments showed that this was an efficient method for sustaining the respective salinity levels in the substrate throughout the entire 21 days experimental period. At four sampling times (0, 7, 14, 21 DAT) six plants (each considered as a single replication) per genotype and treatment were harvested. Growth, agronomical, and physiological parameters were evaluated on three plants; phytochemical indices were determined on the other three.

### Plant growth and developmental characteristics

At each sampling time (0, 7, 14, 21 DAT), individual plant height was recorded from the soil surface to the base of the petiole of the youngest fully expanded leaf. Then, the roots were separated from the shoots, and the shoot fresh weight was determined. The leaves were counted and subsequently removed from the stems for further study. The leaf area (LA) was evaluated from digital images that were acquired using a flatbed scanner. The acquired images were analyzed using ImageJ (Schneider et al., 2012). The samples (leaves and stems) were then oven-dried at 70°C till stable weight and their dry weight was determined.

### Molecular biology

Total RNA isolation was carried out using the Trizol reagent (Chomczynski, 1993). The extracted RNA was subsequently used for RT-PCR using the Takara Prime Script kit. Alternatively, total RNA was extracted from 100 mg of tissue with TRItidy G^TM^ (AppliChem). cDNA was synthesized in a 20 μl reaction mixture containing ±4 μg denatured RNA, 50 pM oligo (dT_17_) primer (VBC-Biotech, Vienna, Austria), 1 mM dNTPs mixture (Qiagen, Germany), 4 μl 5X buffer (PrimeScript, Takara, Shiga, Japan), 20 Units of RNase inhibitor (Biolabs), and 100 Units reverse transcriptase (PrimeScript, Takara, Shiga, Japan). cDNA synthesis conditions were as follows: 5 min at 65°C, 60 min at 42°C, 70°C for 15 min. cDNA samples were diluted 2.5x. PCR was performed in a final volume of 25 μl containing 1.25 units Taq DNA polymerase (Life Technologies, Rockville, Md or Invitrogen, Carlsbad, California), 0.5–6 μl cDNA, 0.3 pM gene—specific primer, 0.3 mM dNTPs mixture (Qiagen), 1.5 mM MgCl_2_ (Invitrogen), and 1x PCR reaction buffer minus Mg^2+^ (Invitrogen). Conditions of PCR amplification were as follows: 2 min at 94°C, 1 min at 92°C, 1 min at the appropriate annealing temperature, 35 s at 72°C, and a final extension step of 5 min at 72°C. The primers used were AGCCCATCTTCCTCTCTGTTC and CCTCAAAGCCAATAGCCA AG for *SPMS*, CCAACCACAACAACGACAAC and TCCAAAACAAGCACCTTTCC for *SPDS*, and GTCTGGTGATGGTGTTAGC and CCTATCAGCAATTCCAGGAAAC for *ACTIN* (33, 33, and 30 cycles, respectively). All amplicons were separated by 1.4% (w/v) agarose gel electrophoresis, stained with EtBr and visualized under UV light. The total density of individual bands was measured with GelEval software package (v1.37, Frog Dance Software).

### Protein extraction and electrophoresis

Proteins were extracted and treated as described in Papadakis and Roubelakis-Angelakis (2005). For native electrophoresis and in-gel assays, proteins were resolved by native PAGE and then stained according to each enzyme.

### Enzymatic assays for NADPH oxidase, polyamine oxidase, catalase, and superoxide dismutase

NADPH oxidase activity was determined as described by Papadakis and Roubelakis-Angelakis (2005). A spectrophotometric method was used for PAO assays (Federico et al., 1985). CAT activity was determined by measuring the initial rates of H_2_O_2_ decomposition at 240 nm (Havir and Mchale, 1987). Total SOD activity was determined using the photochemical assay (Misra and Fridovich, 1977).

### Determination of polyamines

PA titers were determined as described previously (Kotzabasis et al., 1993) using an HP 1100 high-performance liquid chromatographer (Hewlett-Packard).

### Determination of H_2_O_2_ levels

*In situ* accumulation of H_2_O_2_ was detected using the method of Thordal-Christensen et al. (1997). The endogenous levels of H_2_O_2_ content of tissues were determined as described by Sahebani and Hadavi (2009). The content of H_2_O_2_ was estimated by a standard curve.

### Determination of phenolic content and antioxidant capacity

Quantification of total soluble phenol content and antioxidant capacity was performed over a 21 DAT period in WT and transgenic plants treated with 0, 50, 100, 200, and 300 mM NaCl. Approximately 500 mg of leaf material (fifth leaf from the apex) was homogenized at 4°C with 80% MeOH containing 1% HCl. Total soluble phenolic compounds in MeOH leaf extracts were determined using the Folin-Ciocalteu reagent (Chem-Lab NV, Belgium) in a 7.5% Na_2_CO_3_ solution. The extraction mixture was placed in a water bath at 50°C for 5 min and the absorbance was then read at 760 nm with a UV-Vis spectrophotometer (Shimadzu UV-1601). Results were expressed as gallic acid equivalents (GAE) per fresh weight. In addition, total phenolic compounds were determined, qualitatively and quantitatively, in leaf tissues from plants treated for 24 h with 100 mM NaCl. Phenolics were extracted according to Tsiri et al. (2009). All extracts were qualitatively analyzed by Thin Layer Chromatography (TLC) using a stable phase 0.25 mm silica gel 60 and two different mobile phases, nButanol:Acetic-acid:water (3:1:1) and Ethyl Acetate:Acetic acid:Formic acid:water (100:11:11:26). Total phenolic compounds were visualized under UV light after staining with Naturstoff (1% methanolic diphenyl boryl oxyethylamine, followed by 5% ethanolic PEG_4000_). Quercetin dehydrate, rutin and quercetrin were used as standards in all TLC plates. Quantification of total phenolics was performed using the aluminum chloride colorimetric method (Chang et al., 2002). For determining the total antioxidant capacity of leaves, the ferric reducing antioxidant potential (FRAP) assay was used (Benzie et al., 1999) in MeOH leaf extracts prepared similarly to the determination of total soluble phenolic compounds. Absorbance of the mixture was read at 593 nm with a UV-Vis spectrophotometer (Shimadzu UV-1601). Results were expressed as ascorbic acid equivalents (AAE) per fresh weight. All samples were analyzed in triplicates.

### Photosynthetic apparatus-spectroscopic assays

The methods used for measuring extents of energy quenching (qE) component of non-photochemical quenching (NPQ), rates of LEF, and the relative extents of proton motive force (*pmf*) components were as described in Kanazawa and Kramer (2002) except that a newly developed instrument was used. This instrument was based on the non-focusing optics spectrophotometer (NoFOSpec; Kramer and Sacksteder, 1998) but has been modified to allow near-simultaneous measurements of absorbance changes at four different wavelengths, by aiming four separate banks of light-emitting diodes (HLMP-CM15, Agilent Technologies, Santa Clara, CA), each filtered through a separate 5-nm band-pass interference filter (Omega Optical, Brattleboro, VT), into the entrance of a compound parabolic concentrator (Hall et al., 2013). Each complete set of three pulses was deconvoluted by using the procedure described previously to obtain estimates of Electrochromic Shift (ECS; Avenson et al., 2004; Ioannidis et al., 2012a). The instrument was also used to measure changes in chlorophyll *a* fluorescence yield by using the 520-nm light-emitting diode bank as a probe beam. Saturation pulses (>7000 μmol of photons m^−2^s^−1^ PAR) were imposed by using light from the two red actinic LEDs, filtered through heat-absorbing glass. Actinic light was filtered out by using an RG-695 Schott glass filter. Saturation pulse-induced fluorescence yield changes were interpreted as described in Genty et al. (1989). The qE component of NPQ was calculated from the saturation pulse-induced maximum fluorescence yields during steady-state illumination (Fm′) and 10 min (Fm″) after switching off the actinic light (Navakoudis et al., 2007).

### Photosynthetic parameters

Measurements of net photosynthesis (A_*net*_, μmol m^−2^s^−1^), stomatal conductance (Gs, mol m^−2^s^−1^), photosynthetic yield (QY), and chlorophyll content index (CCI) were performed on tobacco plants treated with 0, 50, 100, 200, and 300 mM NaCl over a 21 DAT period. Measurements were performed on the fifth fully expanded leaf (counting from the apex) of 70 days-old plants (time 0), 77, 84, and 91 days-old plants (7, 14, 21 DAT, respectively). Three plants (measurements) were used per genotype, parameter and treatment at each sampling time. Both parameters were monitored using a portable photosynthesis system (LI-6200, LI-COR Inc. Lincon NE, USA). Chlorophyll fluorescence parameters were recorded on the fifth leaf (chosen as above) using a direct portable fluorometer (Photosynthesis yield analyzer MINI–PAM, Walz, Effeltrich, Germany). Photosynthetic yield [effective quantum yield of photochemical energy conversion in PSII or simply quantum yield (QY) was computed in terms of the efficiency of the energy harvesting by open PSII reaction centers in the light as previously described (Kadoglidou et al., 2014). CCI was measured using an Opti-Sciences CCM-200 chlorophyll content meter (OptiSciences Inc., Tyngsboro MA). For both QY and CCI, 10 measurements were taken per genotype and treatment at each sampling day. To avoid photoinhibition, all physiological measurements were performed in a 2 h time span, approximately 3 h after sunrise.

### *In vivo* measurements of proton flux and *pmf* characteristics

Proton Flux and *pmf* can provide useful information on the photosynthetic performance. These analyses were performed by newly introduced techniques that allow us to non-invasively probe the “proton circuit” of photosynthesis. For the theoretical framework for these methods see (Avenson et al., 2004; Ioannidis et al., 2012a and refs therein). We probed the ECS, an index of *pmf* by using a previously described technique called dark-interval relaxation kinetic analysis (Sacksteder et al., 2000), in which steady-state photosynthesis is perturbed by short (up to 0.5 s) dark intervals, allowing the photosynthetic apparatus to relax in ways that reveal information about the system in the steady state. The amplitude of the light–dark ECS signal (ECSt) parameter was obtained by taking the total amplitude of the rapid phase of ECS decay from steady state to its stable level after ~300 ms of darkness (Kanazawa and Kramer, 2002). For the deconvolution, all traces were normalized to the initial dark value (i.e., before actinic) and then the following equation was used (Ioannidis et al., 2012a): ECS_520_ = A_520_−0.5 × A_535_−0.5 × A_505_.

### Fluorescence measurements *in vivo*

NPQ was determined in samples exposed to actinic light of 110 μmol photons m^−2^s^−1^ using PAM-210 fluorometer (Heinz Walz, Germany). Samples were incubated in the dark for at least 10 min prior to measurement. The NPQ-parameter was calculated according to the equation: NPQ = (FM−FM′)/FM′ (Bilger and Björkman, 1990). For the fluorescence induction measurements, the portable Plant Efficiency Analyzer, PEA (Hansatech Instruments) was used as previously described (Kotakis et al., 2014). The method is based on the measurement of a fast fluorescent transient with a 10 μs resolution in a time span of 40 μs–1 s. Fluorescence was measured at a 12 bit resolution and excited by three light-emitting diodes providing an intensity of 3000 μmol photons m^−2^s^−1^ of red light (650 nm). For the estimation of Ka, DCMU inhibited electron transport after QA and analysis of the area closure was performed according to the method of Melis as modified (Ioannidis et al., 2009).

### Determination of ions

Measurements of ion content in soil and in plants treated with 0, 50, 100, 200, and 300 mM NaCl were performed over a 21 days period. In addition, electrical conductivity (EC_*se*_) and sodium adsorption rate (SAR) in substrates were determined (Supplemental Table 1). On each sampling day, soil substrates (i.e., the peat: perlite mixture) from three pots per genotype and treatment were dried at 70°C until constant weight. The dried substrates were then water saturated and the pH of the created pastes measured. Thereafter, aqueous saturation extracts were obtained through vacuum filtration and EC_*se*_, water soluble K^+^, Na^+^, Ca^2+^, Mg^2+^, and SAR were determined (Rhoades, 1996). Ion content (K^+^, Na^+^, Ca^2+^, and Mg^2+^) was determined following NaCl treatment. Shoot samples (0.5–1 g) were burnt in a muffle furnace at 500°C for at least 4 h and the residue was dissolved in a 3.6 M HCl, 1.4 M HNO_3_ aqueous solution. Monovalent ions were determined using a flame photometer (Jenway PFP 7, Gransmore Green, Felsted England), and the divalent using an atomic absorption spectrophotometer (ShimadzuAA 6300, Japan).

### Statistical analyses

Wherever applicable, the experiments were designed at a completely randomized pattern in a split-split-plot arrangement, with the genotype, the NaCl dosage and the sampling time as independent factors. Each combined factor had three replicates in all parameters except for the CCI and QY that had 10 replicates. The analysis of variance (ANOVA) and the Tukey's test on the results obtained over the entire experimental period (up to 21 DAT) were performed by the statistical package SPSS (version 22). Duncan's test was done using SigmaPlot 12.0. Student's test was done using JMP (SAS, version 11 pro). Multiple average comparisons, graphs and data processing were performed in EXCEL. Fisher's Protected LSD procedures were used to determine statistical differences between genotypes and treatments at a 5% significance level.

## Results

### The AS-*SAMDC* plants show altered polyamine metabolism

**Figure 2:**
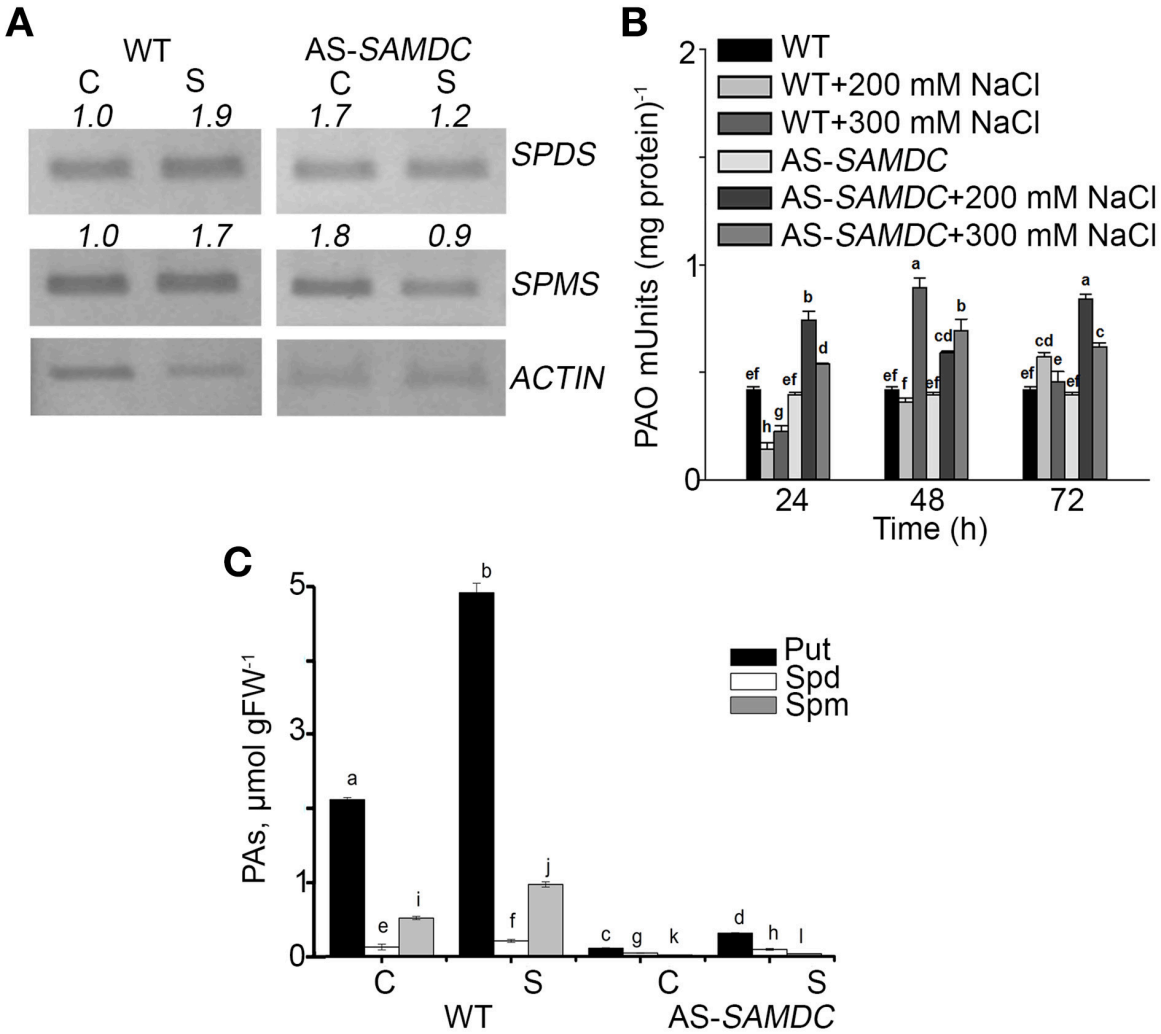
**Polyamine homeostasis in WT and AS-*SAMDC* plants under control and NaCl treatment. (A)** Semi-quantitative RT-PCR analysis of *SPMS* and *SPDS* in control (C) or 100 mM NaCl (S) conditions. Numbers indicate pixel intensity of corresponding bands normalized according to *ACTIN*. **(B)** PAO activity in control and 24, 48, and 72 h post-treatment with 200 or 300 mM NaCl. Data are means ± SE of three biological replicates with three technical replicates each. **(C)** PA levels in WT and AS-*SAMDC* plants in control **(C)** or 200 mM NaCl (S). Data are means ± SE of three biological replicates and different letters indicate significant difference (Student's *t*-test, *P* < 0.05).

Previously, we used young transgenic seedlings of AS-*SAMDC* and showed that NaCl stress can be used to monitor the effects of altered ratio of biosynthesis/catabolism of PAs and also the contribution of PAO-generated H_2_O_2_ in the adaptation of plants to stress and in developmental processes (Moschou and Roubelakis-Angelakis, 2014). We estimated the mRNA abundance of the PA biosynthetic enzymes Spd synthase (*SPDS*) and Spm synthase (*SPMS*) in control and NaCl treated WT and AS-*SAMDC* plants. Under control conditions (0 mM NaCl), mRNA levels of *SPDS* and *SPMS* were significantly higher in AS-*SAMDC* plants by almost two-fold compared to WT plants. During NaCl stress (100 mM), both *SPDS* and *SPMS* mRNA levels increased in WT (ca two-fold), while they significantly decreased in AS-*SAMDC*, when compared to their corresponding controls (Figure 2A). Under control conditions, WT and AS-*SAMDC* exhibited similar PAO activity. Twenty four hours post-treatment with 200 mM NaCl, PAO activity decreased by 60% in WT whereas increased by 80% in the AS-*SAMDC* plants. In the WT it increased by 20% and in AS-*SAMDC* by two-fold 72 h post-treatment (Figure 2B). Following a decrease at 24 h (50%), PAO activity was transiently increased (two-fold) in WT 48 h post-treatment with 300 mM NaCl, while at 72 h it was similar to the corresponding control levels. In AS-*SAMDC* at 300 mM NaCl PAO showed a constant increase.

Next, we determined the titers of endogenous PAs in WT and AS-*SAMDC* grown in perlite. PAs were significantly lower in AS-*SAMDC*, in agreement with previous results (Figure 2C; Moschou et al., 2008b). WT contained higher titers of Put (four-fold), Spm (two-fold), and Spd (20%) compared to the *AS-SAMDC*. NaCl resulted to a significant increase in PAs in WT (107, 20, and 46% increase in Put, Spd and Spm, respectively, whereas in AS-*SAMDC* only Put increased (29%); Spd and Spm titers did not change (Figure 2C).

### Depletion of SAMDC results to increased biomass under control conditions but reduced biomass under NaCl

**Figure 3:**
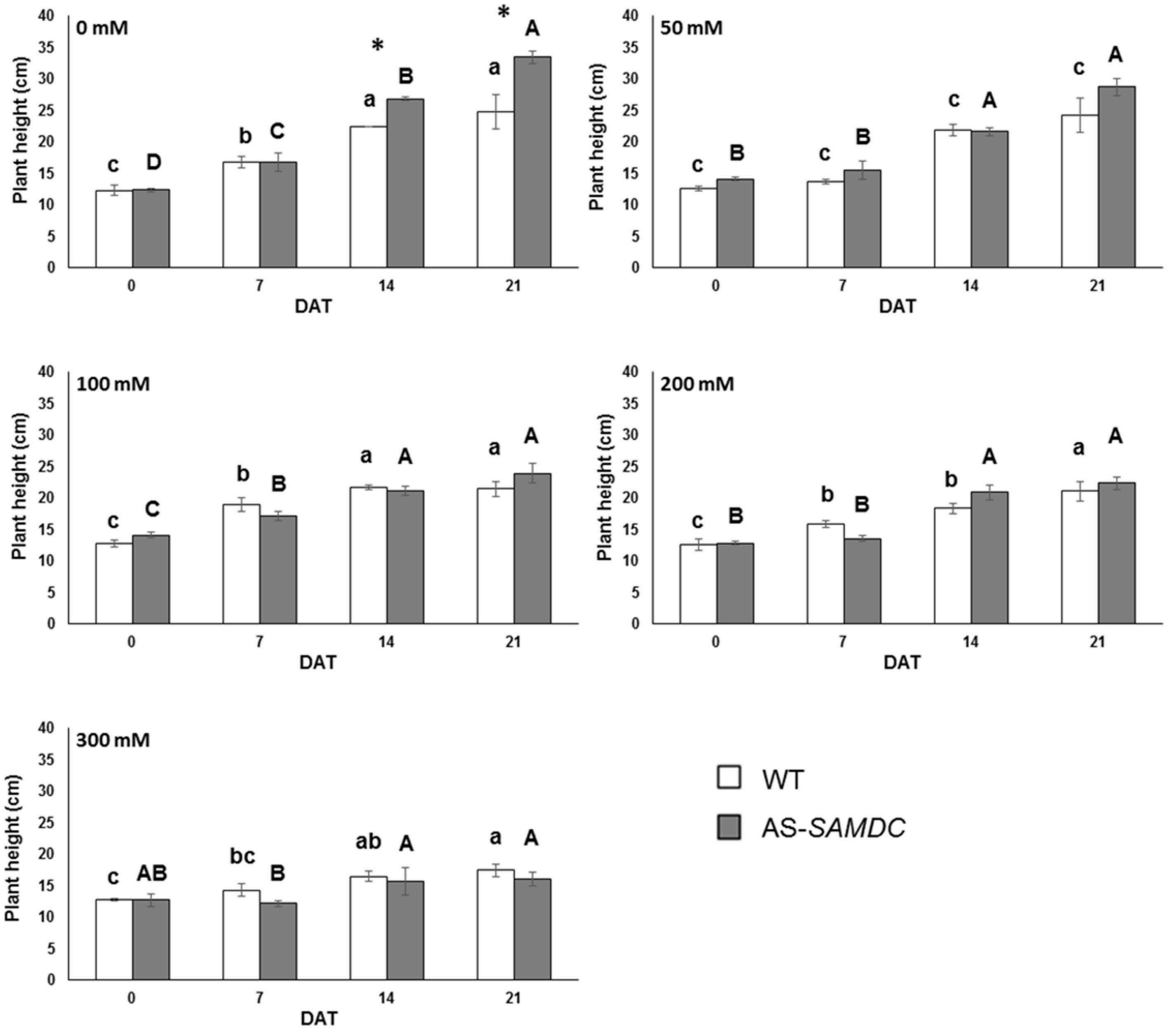
**Plant height of WT and AS-*SAMDC* plants under control and NaCl treatment**. Plant height in cm at different NaCl concentrations (0, 50, 100, 200, 300 mM), 0, 7, 14, and 21 DAT. Data are means ± SE. Different letters (lower case for WT, upper case for AS-*SAMDC*) indicate significant differences over time within the same genotype, based on Tukey's HSD test (*P* < 0.05). Asterisks indicate significant difference of mean values of AS-*SAMDC* from WT (Student's *t*-test; ^*^P < 0.05).

We examined the effect of SAMDC depletion on the development and NaCl stress tolerance. The ANOVA test indicated that all developmental parameters examined were significantly (*P* ≤ 0.001) affected by sampling time and by the five different NaCl concentrations. In most cases, the effect of the genotype, and the effect of two-way interactions on the various parameters were also significant (Supplemental Table 2). In order to facilitate comparisons, we examined either the effects of each NaCl treatment over a 21 DAT, or the means across the four sampling time points were averaged per NaCl concentration over the entire experimental period (up to 21 DAT). The latter is exemplified throughout Supplemental Figures. Control AS-*SAMDC* were on average 1.2-fold taller than the respective WT over the 21 days experimental period (Supplemental Figure 1A). Under NaCl stress, AS-*SAMDC* plants were less resilient and an abrupt decrease of their height was observed (Figure 3; Supplemental Table 2; genotype × NaCl interaction, *P* < 0.05). At the highest NaCl concentration (300 mM), plant height was reduced by 52% in AS-*SAMDC* plants, and only 29.5% in WT plants, compared to the respective control plants (0 mM) at the same time point (Figure 3; 21 DAT). Similar trend was observed at lower NaCl concentrations (100 and 200 mM). The dose-response curves over the whole experimental period (Supplemental Figure 1A) show that only the highest concentration of NaCl (300 mM) affected the height of WT while all NaCl concentrations negatively affected the height of AS-*SAMDC.* NaCl stress caused a reduction in leaf area per plant (LA; cm^2^) in both genotypes (Supplemental Table 2; Supplemental Figure 2), which was greater in AS-*SAMDC* (Supplemental Figures 1B, 2). This reduction in AS-*SAMDC* (reaching a 42% decrease) was evident at all NaCl concentrations, whilst in WT only at the highest ones (Supplemental Figure 1B). Control AS-*SAMDC* had significantly higher number of leaves throughout the experimental period (Supplemental Figure 1C). NaCl stress led to a significant decrease in leaf number regardless genotype (Supplemental Table 2). Although AS-*SAMDC* had more leaves at all NaCl concentrations, their leaf number abruptly declined upon NaCl treatment (Supplemental Figure 1C).

**Figure 4:**
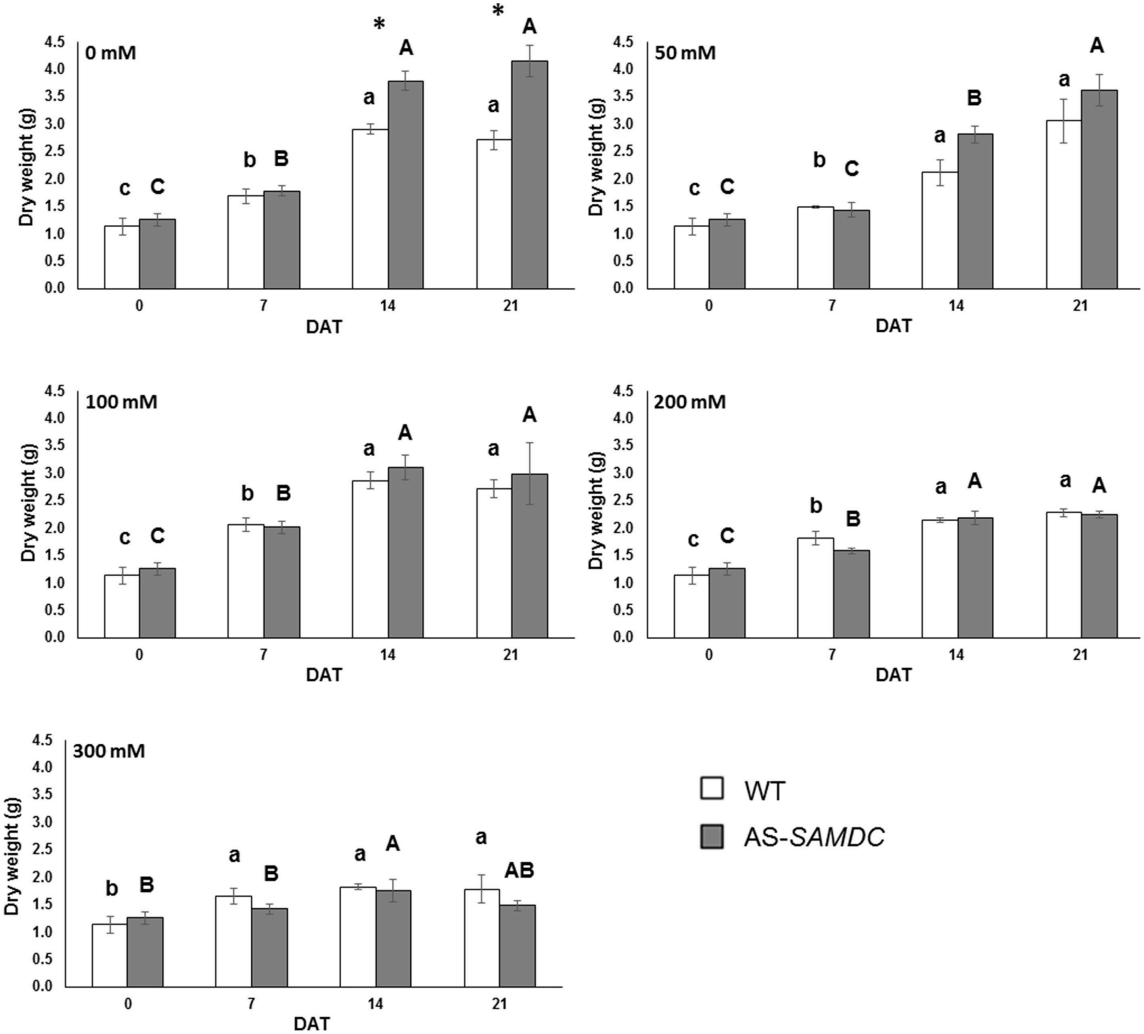
**Dry weight of WT and AS-*SAMDC* plants under control and NaCl treatment**. Dry weight (g) at different NaCl concentrations (0, 50, 100, 200, 300 mM), 0, 7, 14, and 21 DAT. Data are means±SE. Different letters (lower case for WT, upper case for AS-*SAMDC*) indicate significant differences over time within the same genotype, based on Tukey's HSD test (*P* < 0.05). Asterisks indicate significant difference of mean values of AS-*SAMDC* from WT (Student's *t*-test; ^*^P < 0.05).

Generally, the above ground fresh weight (FW) of controls was not significantly different between the two genotypes neither at 0 DAT nor during the experimental period (Supplemental Figure 3). NaCl resulted to a reduction of FW, yet this effect was similar in both genotypes (Supplemental Table 2; genotype × NaCl interaction). Notably, the highest NaCl concentrations affected more the FW of AS-*SAMDC* (65 vs. 57% in WT compared to controls) at 21 DAT (Supplemental Figure 3). Interestingly, when the above ground biomass was expressed as dry weight (DW), the control AS-*SAMDC* plants showed increased biomass compared to the WT from 14 DAT and onwards (Figure 4; 25%). NaCl significantly impaired DW in both genotypes but to a different extent (Figure 4; Supplemental Table 2; genotype × NaCl interaction, *P* < 0.001; Supplemental Figure 4). The same trend was observed at 50 mM NaCl even though the differences were not statistically significant. At high NaCl concentrations however, a trade-off between AS-*SAMDC* DW and NaCl tolerance was evident (a 64% decrease in AS-*SAMDC* at 300 mM, and a 34% decrease in WT at 300 mM Figure 4).

### Depletion of SAMDC halves maximal photosynthesis in high salt

**Figure 5:**
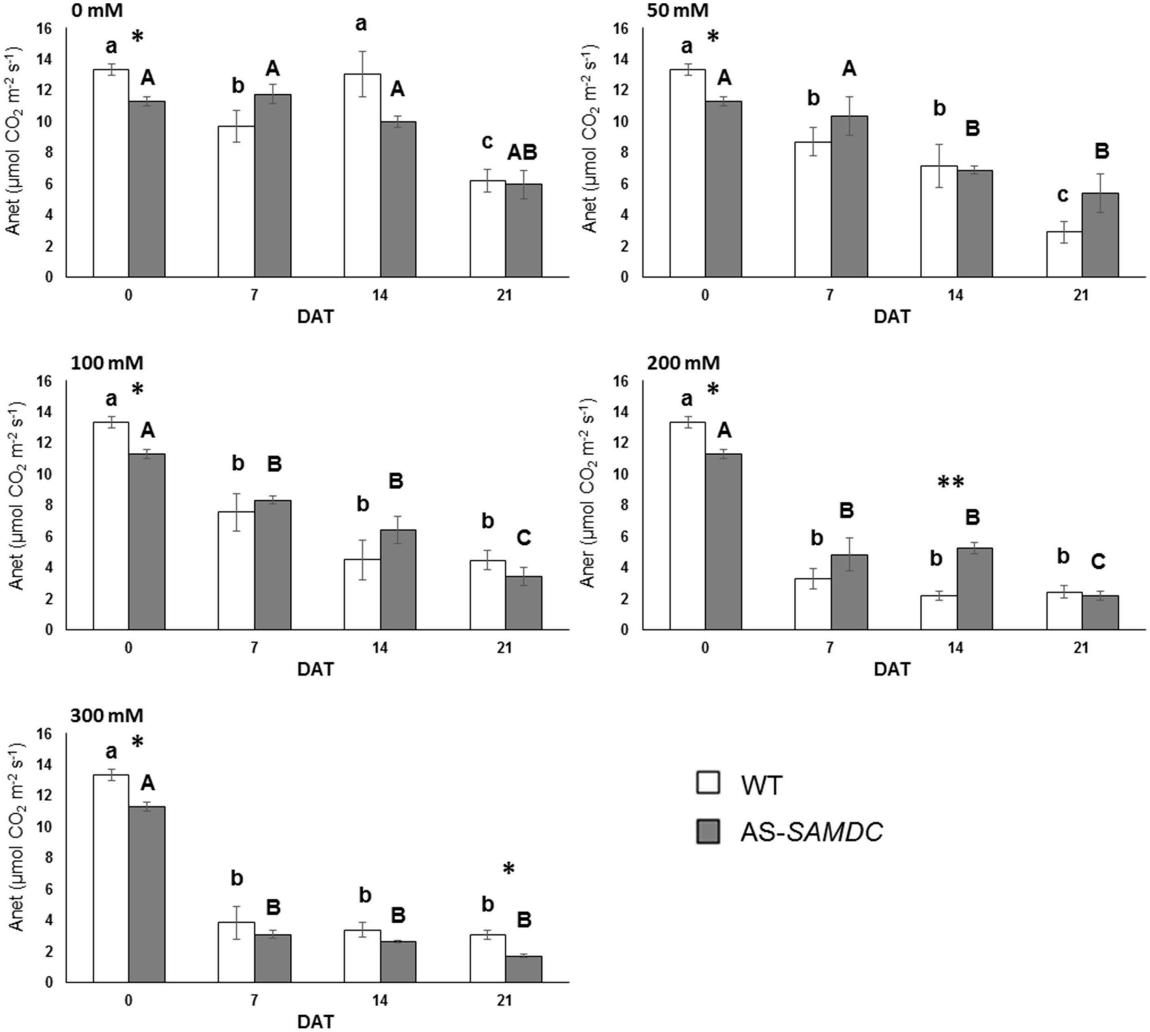
**Net photosynthesis of WT and AS-*SAMDC* plants under control and NaCl treatment**. Net photosynthesis (μmol CO^2^ m^−2^s^−1^) at different NaCl concentrations (0, 50, 100, 200, 300 mM), 0, 7, 14, and 21 DAT. Data are means ± SE. Different letters (lower case for WT, upper case for AS-*SAMDC*) indicate significant differences over time within the same genotype, based on Tukey's HSD test (*P* < 0.05). Asterisks indicate significant difference of mean values of AS-*SAMDC* from WT (Student's t-test; ^*^P < 0.05 and ^**^P < 0.01).

Considering the increased DW of AS-*SAMDC* plants under normal growth conditions, we examined whether this correlated to increased photosynthetic potential. We assessed maximal photosynthetic rates in terms of CO_2_ fixation for the 21 DAT period. Net photosynthetic rate, A_net_, expressed as the rate of net CO_2_ assimilated per leaf area was severely impaired by NaCl in both genotypes regardless of the concentration used (Figure 5; Supplemental Table 2). Interestingly though, at 21 DAT AS-*SAMDC* showed ca. 50% lower A_*net*_ at 300 mM NaCl compared to WT (AS-*SAMDC* 1.6 vs. 3.2 mol m^−2^m^−1^ for WT). At the same time point, the highest NaCl concentration affected more A_*net*_ of AS-*SAMDC* (up to 72%) than of WT (up to 50%) compared to corresponding controls. On the other hand, at lower NaCl concentrations (e.g., 50 mM) or earlier time points (e.g., 200 mM, 14 DAT), an inverse trend could be observed. Stomatal conductance (mol m^−2^s^−1^) was not significantly affected by NaCl, genotype or the interaction between these two factors (Supplemental Table 2), although the AS-*SAMDC* showed in some cases a slightly reduced response under NaCl (Supplemental Figure 5). Generally, WT and AS-*SAMDC* controls showed comparable values of QY (Supplemental Figure 6). Yet, NaCl slightly reduced QY especially at concentrations over 200 mM, regardless of the genotype, but to a different extent. In particular, at 21 DAT the reduction in QY compared to control plants was greater in the NaCl-treated AS-*SAMDC* compared to WT plants at the highest NaCl concentration (a maximal 4.4% decrease vs. 1.3%, respectively), suggesting that SAMDC has a role in sustaining the efficiency of light use in Photosystem II (PSII) during photosynthesis.

**Figure 6:**
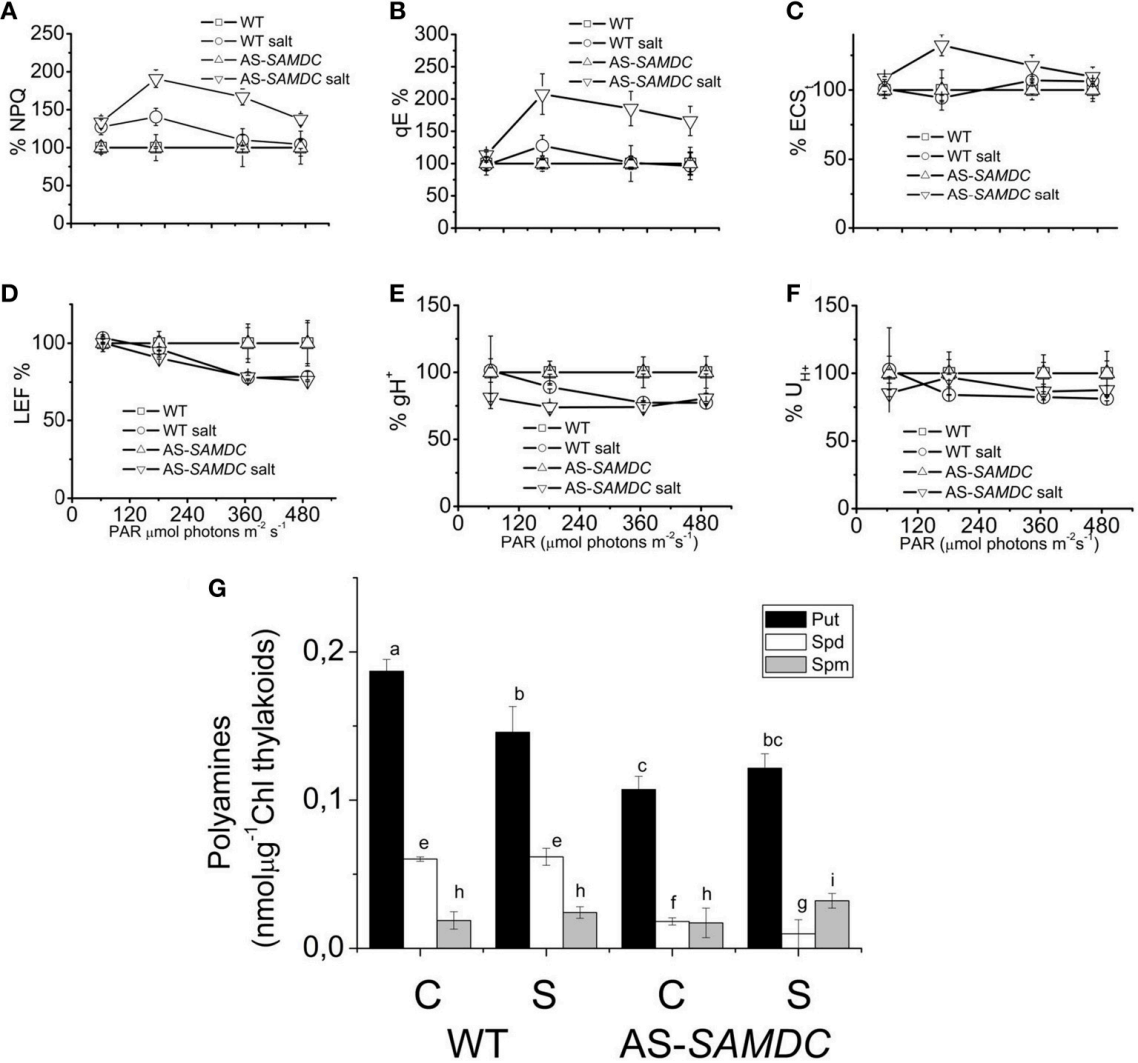
**Photosynthetic parameters and PA titers in WT and AS-*SAMDC* plants grown in perlite 24 h post-treatment with 200 mM NaCl at different light intensities. (A)** Non-photochemical quenching (NPQ) at four different light intensities (0, 64, 181, 366, and 490 μmol photons m^−2^s^−1^). **(B)** High energy quenching (qE). **(C)** Light-induced proton motive force estimated as electrochromic shift in thylakoids. **(D)** Linear electron flow (LEF) at four different light intensities. **(E)** Apparent conductivity of the thylakoidal ATPase to protons (gH+). **(F)** Total proton flux from lumen to stroma (U_H+_). Data in **(A–F)** are means ± SE (*n* > 3). Error bars denote standard error (*n* > 3). Steady state photosynthesis in WT and AS-*NtSAMDC* estimated in intact plants treated with high dose of NaCl and control plants. **(G)** Thylakoidal PA levels. Data are means ± SE of three biological replicates. Different letters indicate significant difference (Student's *t*-test, *P* < 0.05).

Next, we examined in more detail the photosynthetic apparatus in seedlings grown under more defined conditions (i.e., growth in perlite with MS). We determined the photo-protection capacity *via* NPQ, and the qE at 200 mM NaCl at four different light intensities. These mechanisms are employed by cells to dissipate the adverse effects imposed by high light intensity (Figures 6A,B; and Supplemental Table 4). NPQ is the sum of the total effect of high qE, state transitions, and photoinhibition (pI). At low light intensities NaCl affected slightly the NPQ and qE (about 27% in WT and 33% in AS-*SAMDC* compared to controls). However, with increasing light intensity the differences between WT and AS-*SAMDC* increased (NPQ by 40 and 91%, respectively). By estimating qE values under these conditions we confirmed that the effect in NPQ is largely due to qE and not to qI or state transitions. NaCl led to a profound increase of qE only in the AS-*SAMDC* (207 vs. 27% in WT; Figure 6B). In addition, Electrochromic Shift (ECS), an index of *pmf* was determined. The techniques used take advantage of the ECS (known also as Δ^520^ or ΔA^518^) of certain carotenoid species that naturally occur in thylakoidal membranes. The ECS is a linear indicator of changes in *trans* thylakoid ΔΨ and is particularly useful since it responds to the *trans* thylakoid movement of protons, as well as other charged species. ECS was significantly increased in AS-*SAMDC* (Figure 6C). In particular, NaCl exerted a mild effect, if any, on WT whereas the same NaCl concentration increased thylakoidal *pmf* of AS-*SAMDC* (by 32%). On the other hand, NaCl hindered linear electron flow (LEF) at higher light almost at the same level for both genotypes (21% in WT and 25% AS-*SAMDC*, Figure 6D). The apparent conductivity of the thylakoidal ATPase was much more sensitive in AS-*SAMDC* (about 19% at low light) than in WT (practically similar values of gH^+^ between 0 and 200 mM NaCl; Figure 6E). Only at higher light intensities, there was a clear decrease in gH^+^ values in WT. NaCl decreased the total proton flux (U${}_{H}^{+}$) from lumen to stroma (19% in WT and up to 23% in AS-*SAMDC*, Figure 6F). Furthermore, these changes were associated with significant reductions of Put and Spd in AS-*SAMDC* (Figure 6G).

The leaves of AS-*SAMDC* were significantly greener at the start of the experiment (0 DAT, 0 mM NaCl), as indicated by the significantly higher CCI at pre-treatment (Supplemental Figure 7). Yet, this difference between controls of the two genotypes became non-significant when examined throughout the experimental period. Interestingly, at 21 DAT, NaCl treatment significantly increased CCI in both genotypes, compared to controls, an increase becoming evident from 100 mM NaCl onwards in WT, but only at the highest NaCl concentration in the AS-*SAMDC* plants (Supplemental Figure 7).

### WT and AS-*SAMDC* transgenic tobacco plants accumulate comparable levels of hydrogen peroxide

**Figure 7:**
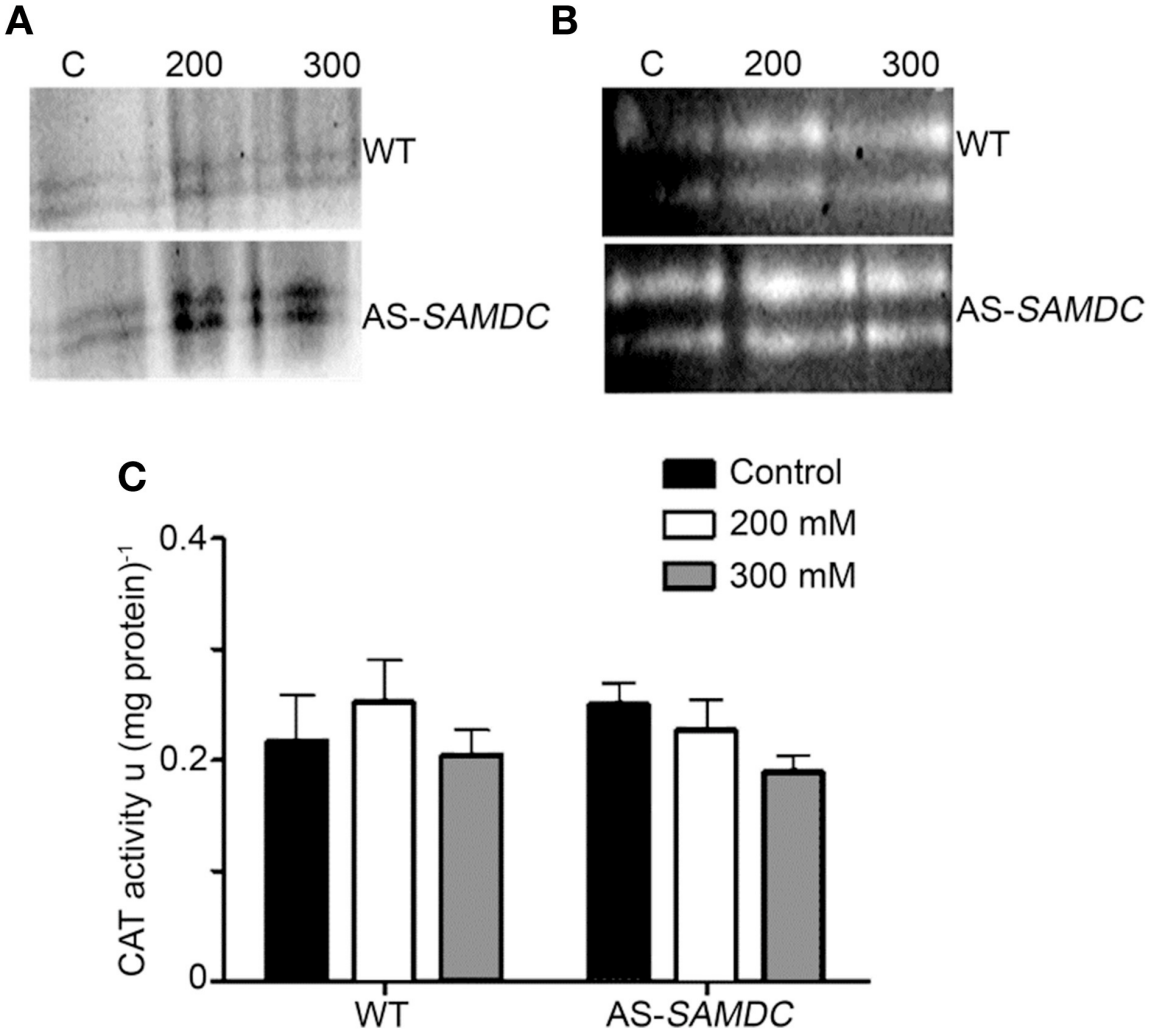
**Activities of NADPH-oxidase, SOD and CAT in WT and AS-*SAMDC* plants under control and 24 h post-treatment with 200 or 300 mM NaCl. (A)** In-gel activity assay of NADPH-oxidase. Images are from a single representative experiment replicated three times. C, control. **(B)** In-gel activity assay of cytoplasmic (CuZn-SOD; upper band) or mitochondrial SOD (Mn-SOD; lower band). Images are from a single representative experiment replicated three times. C, control. **(C)** Specific activity of CAT. Data are means ± SD of three biological replicates.

Considering the paramount role of PAs in the regulation of H_2_O_2_ homeostasis (Andronis et al., 2014), and the effect of ROS in plant development and stress defense, we examined main oxidant/antioxidant machineries and H_2_O_2_ titers in WT and AS-*SAMDC* control or stressed plants. Hydrogen peroxide and superoxides (O${}_{2}^{.-}$) are tightly inter-dependent in cells and elaborate machinery regulates the balance between these two ROS. One of the major oxidative machineries (i.e., ROS producers) is that of the plasma membrane localized nicotinamide adenine dinucleotide phosphate-oxidase (NADPH-oxidase) that produces O${}_{2}^{.-}$. The mRNA abundance of the NADPH-oxidase subunits, the respiration burst homolog *rbohD* increased in the AS-*SAMDC* control plants, while that of *rbohF* was reduced under control conditions (Supplemental Figure 8; *P* < 0.05). NaCl induced the accumulation of *rbohD* and *rbohF* mRNA in both genotypes in a dose-dependent manner, with WT plants showing higher induction. On the other hand, NADPH-oxidase activity in AS-*SAMDC* was significantly higher under both control and NaCl conditions (Figure 7A).

NADPH-oxidase feeds superoxide dismutase (SOD) in the cytoplasm, mitochondria and chloroplasts with O${}_{2}^{.-}$, to dismutate it to H_2_O_2_. We observed a slight decrease of *MnSOD* mRNA levels in AS-*SAMDC* controls, while in both WT and AS-*SAMDC* mRNA levels of *MnSOD* were further decreased (Supplemental Figure 8). *CuZnSOD* mRNA levels were significantly higher in AS-*SAMDC* compared to WT, during control and NaCl conditions (Supplemental Figure 8). On the contrary, we observed increase of SOD isoforms and activity in both WT and AS-*SAMDC* (Figure 7B; Supplemental Figure 8). These results suggest that increased O${}_{2}^{.-}$ in AS-*SAMDC* is compensated by SOD controlled at the post-transcriptional level.

**Figure 8:**
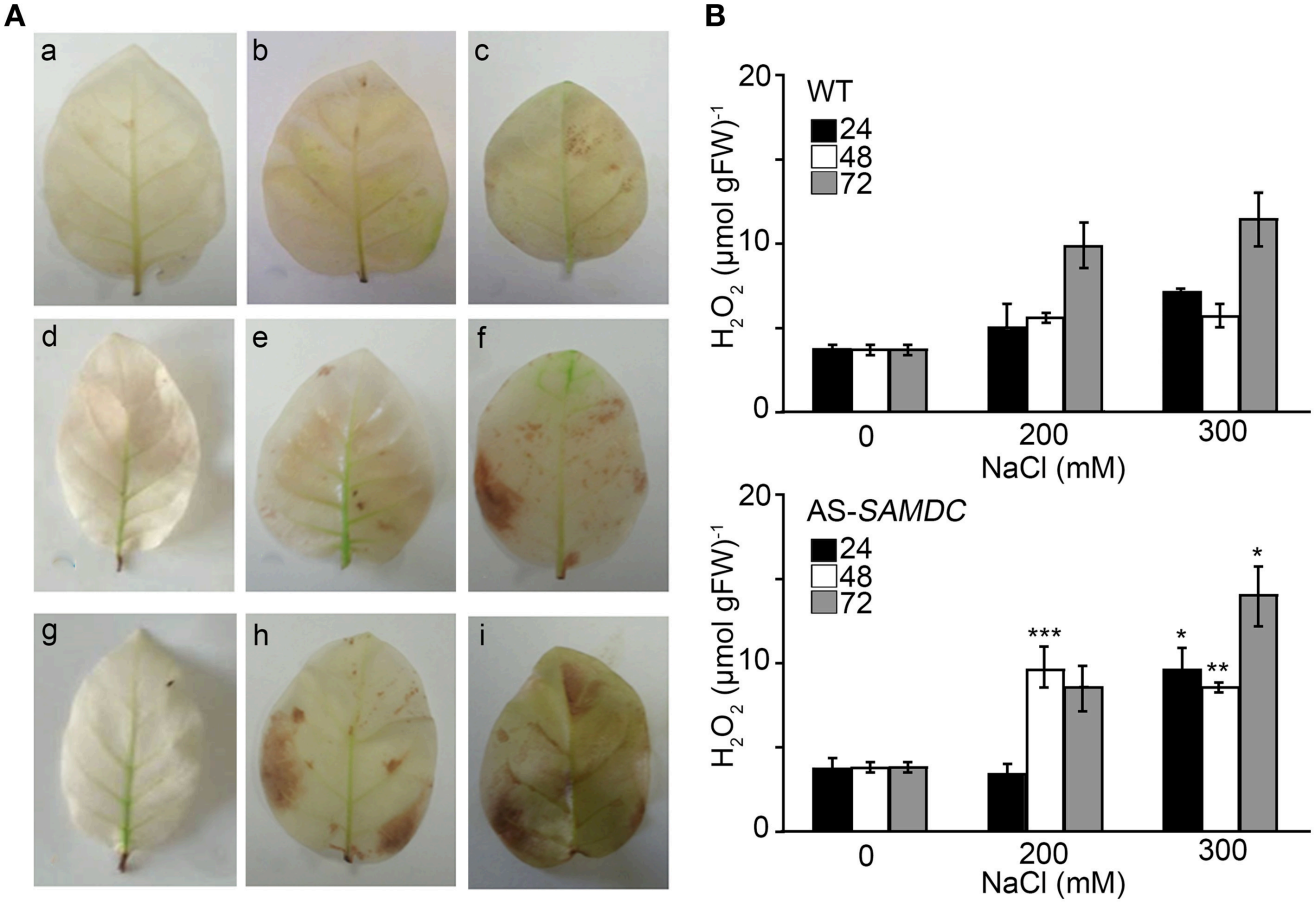
**Levels of Hydrogen peroxide in WT and AS-*SAMDC* leaves under control and NaCl treatment. (A)** *In situ* detection of H_2_O_2_ leaves of WT and AS-*SAMDC* plants after exposure to 200 and 300 mM NaCl; 24 h (a–c), 48 h (d–f), and 72 h (g–i), control (a,d,g); 200 mM NaCl (b,e,h); 300 mM NaCl (c,f,i). Images are from a single representative experiment replicated three times. **(B)** Hydrogen peroxide levels in WT and AS-*SAMDC* control plants. Data are means ± SD of three biological replicates. Asterisks indicate significant difference of mean values of AS-*SAMDC* from WT (Student's *t*-test; ^*^P < 0.05, ^**^P < 0.01, and ^***^P < 0.001).

Catalase (CAT) is the main enzyme that scavenges bulk H_2_O_2_. We observed a small non-significant increase of CAT activity in control AS-*SAMDC*, and a decrease of the corresponding levels during NaCl stress when compared to the WT (Figure 7C) and marginally increased H_2_O_2_ titers in leaves (Figures 8A,B). On the contrary, under NaCl AS-*SAMDC* accumulated significantly more H_2_O_2_ than WT.

**Figure 9:**
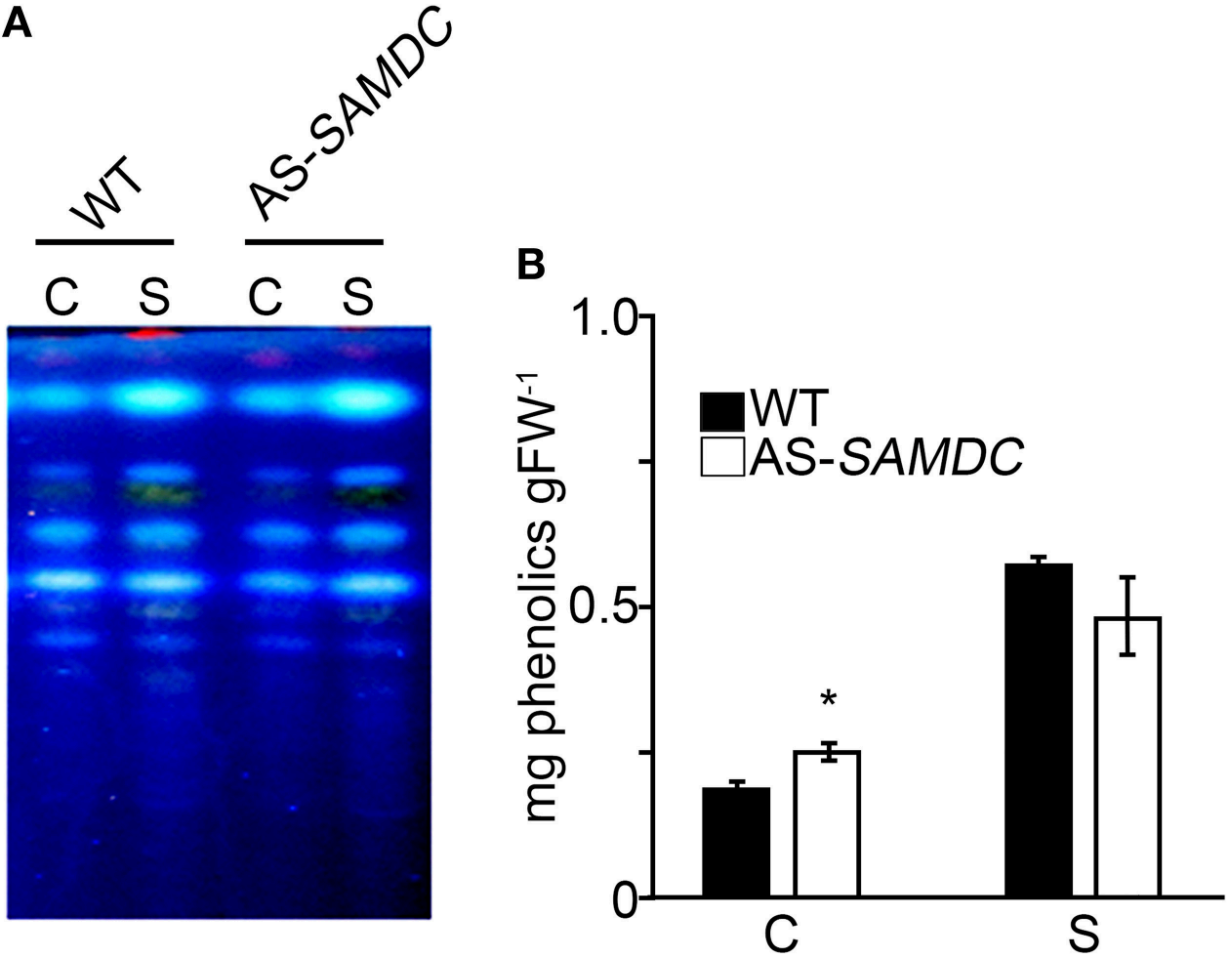
**Qualitative and quantitative analysis of total phenolics from WT and AS-*SAMDC* transgenics. (A)** TLC analysis and **(B)** Quantification of total phenolics from plants 24 h post-treatment with 100 mM NaCl. Data are means ± SE of three biological replicates. Asterisk indicate significant difference of mean values of AS-*SAMDC* from WT (Student's *t*-test; *P* < 0.05).

Since antioxidant defense of plants is also complemented by non-enzymatic antioxidants, such as phenolics, we examined their content of 24 h post-treatment with 100 mM NaCl (phenolics; Figure 9A), or over a period of 21 DAT (phenolics and antioxidant capacity; Supplemental Figure 9). Total phenolics were significantly higher in the AS-*SAMDC* under control and NaCl. No significant alterations in the zonation pattern between WT and AS-*SAMDC* plants were found (Figure 9A). Upon NaCl treatment, both the WT and AS-*SAMDC* showed significant increase in total phenolics (two- to three-fold; Figure 9B). These results were further confirmed in NaCl-treated plants over a period of 21 DAT, when significantly higher content of total soluble phenolic compounds of AS-*SAMDC* plants *versus* WT at 100 mM NaCl was reconfirmed (20% higher), and further recorded at 300 mM NaCl (15% higher; Supplemental Figure 9A). On the contrary, no particular difference in antioxidant capacity was observed between genotypes at control conditions nor upon NaCl treatment (Supplemental Figure 9B).

### The AS-*SAMDC* plants accumulate less K^+^, Ca^2+^, and Mg^2+^ and equal Na^+^ compared to WT under control conditions

**Figure 10:**
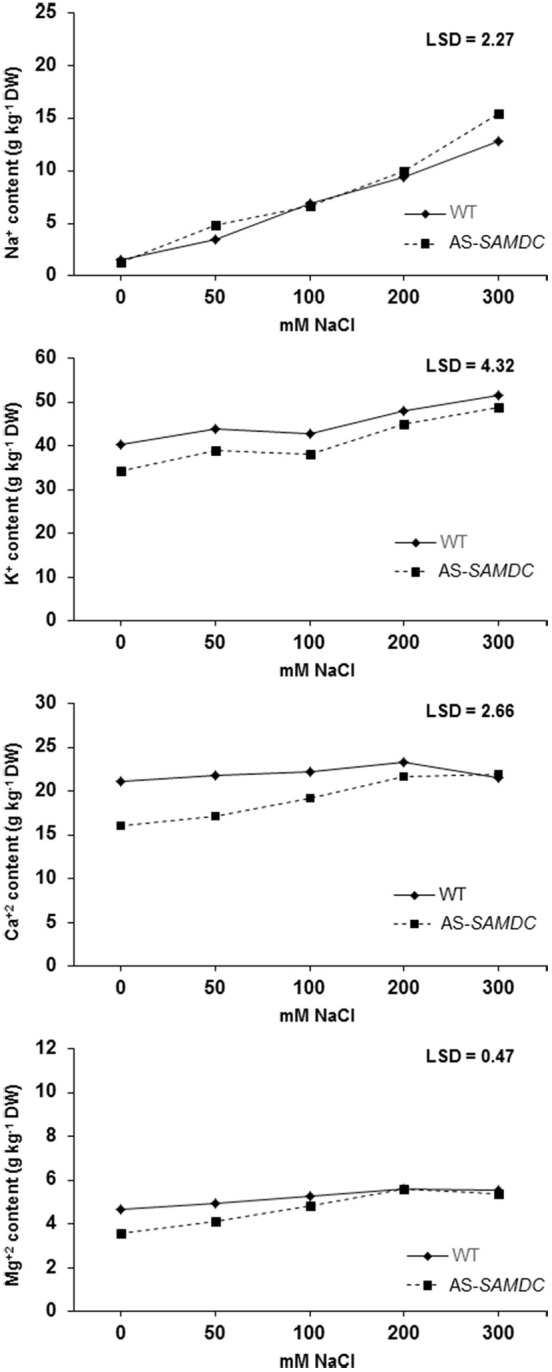
**Ion accumulation in the leaf tissue of WT and AS-*SAMDC* under control and NaCl treatment**. Accumulation of Na^+^, Cl^−^, Ca^2+^, Mg^2+^ in the leaves of WT and AS-*SAMDC* plants at different NaCl concentrations (0, 50, 100, 200, 300 mM). Means are averaged over the whole experimental period, and each data point corresponds to the average of 9 observations [i.e., 3 replications per genotype per sampling date (7, 14, and 21 DAT)]. Fisher's Protected LSD procedures was used to determine statistical differences between genotypes and treatments at a 5% significance level.

PAs play a major role in controlling ion homeostasis (Shabala and Pottosin, 2014), and thus we examined whether AS-*SAMDC* showed imbalances in ion accumulation that could drive NaCl sensitivity. Under control conditions, at the start of the experiment, WT and AS-*SAMDC* showed similar ion content in leaves (Supplemental Table 3). Thereafter, leaves of WT contained higher amount of K^+^, Ca^2+^, and Mg^2+^ than those of AS-*SAMDC* (Figure 10). During NaCl treatment, increasing NaCl concentrations caused in some cases statistically significant increase in ion accumulation. In particular, Na^+^ content in leaves increased in both genotypes in a dose-dependent manner over the experimental period (Figure 10A). Statistically significant differences between the two genotypes were observed only at 300 mM NaCl (AS-*SAMDC* leaves accumulated 16% more Na^+^ than WT ones). Na^+^ content in stressed WT and AS-*SAMDC* plants at 300 mM NaCl was ca. 5- and 12-fold higher, respectively compared to the corresponding controls. Leaves of WT contained higher K^+^ content throughout the experimental period compared to AS-*SAMDC* (Figure 10B). Increasing NaCl concentrations led to increased leaf K^+^ content, regardless of the genotype (at 200 and 300 mM). The pattern of Ca^2+^ accumulation was different between the genotypes (Figure 10C). Control WT plants contained 1.3-fold more Ca^2+^ in leaves than AS-*SAMDC*. Interestingly, NaCl did not affect leaf Ca^2+^ content in WT, where a rather constant content of Ca^2+^ was maintained, whereas NaCl significantly increased Ca^2+^content in AS-*SAMDC* (over 35% at >200 mM NaCl). Similarly to Ca^2+^, Mg^2+^ content was 1.3-fold higher in the leaves of WT compared to the AS-*SAMDC* control plants (Figure 10D). The response of Mg^2+^ accumulation under NaCl conditions followed a similar pattern to Ca^2+^, although the increase of Ca^2+^ content in the latter case was more prominent in the AS-*SAMDC* plants.

## Discussion

PAs have one agreed-on function, to protect cells in adverse environments or when challenged by biotic factors (Alcazar et al., 2006; Hatmi et al., 2015), and high PAs content during stress has been associated with increased stress tolerance. Genetic enhancement of the PA biosynthetic (Capell et al., 2004; Kasukabe et al., 2004) or the decrease of PA catabolic pathway (Moschou et al., 2008b; Moschou and Roubelakis-Angelakis, 2014), resulted to plants with higher PAs content and increased tolerance against abiotic stress. However, the molecular roles of PAs remain rather conjectural and their pleiotropic effects call for careful dissection of causal relationships with the observed phenotypes.

AS-*SAMDC* show decreased PAs biosynthetic rate without significant phenotypical alterations at early stages of development. Therefore, AS-*SAMDC* represent an excellent genetic tool to study PA functions during stress. PA titers in AS-*SAMDC* were significantly lower at the developmental stage used, than in the WT (Figure 2; Moschou et al., 2008b). We should note that plants grown in soil may absorb PAs from the substrate and increase their turnover (Chibucos and Morris, 2006), which in turn may alleviate the effects of the decreased PA titers in AS-*SAMDC*, thereby buffering possible decreases in vigor. However, we did not observe significant phenotypical differences when we grew AS-*SAMDC* on PA-free substrates (although absorbance changes at 535 nm *in vivo* were striking; see ECS calculations). The decrease of Put levels may look inconsequent with the function of SAMDC in producing solely higher PAs. However, Put decrease in AS-*SAMDC* might be due to decreased fuelling of Spd and Spm to the cytoplasmic and peroxisomal PAOs that produce Put (Moschou et al., 2008c). Furthermore, the observed compensatory increase of *SPDS* and *SPMS* mRNA abundance was not sufficient to achieve restoration of PA titers to WT levels. Total PAO activity did not significantly change under control conditions in AS-*SAMDC*. Interestingly, during NaCl stress, SAMDC depletion could drive a decrease of SPDS and SPMS levels, and a significant increase of PAO. The previous amendments are expected to further decrease the PA biosynthesis/catabolism ratio in AS-*SAMDC*. Our results point out that the low ratio of PA biosynthesis/catabolism is not sufficient to trigger cell death in the absence of additional signals (such as NaCl stress in our case). Perhaps, modules that are controlled by this ratio and feedback to cell death can be activated when this ratio gets very low.

Unlike our previous study in which we used young AS-*SAMDC* seedlings, here we examined growth parameters of AS-*SAMDC* over a 90 days period. Interestingly, and against all expectations, AS-*SAMDC* outperformed WT in DW biomass accumulation (>30%; 21 DAT) under control conditions accompanied by altered developmental characteristics, such as increased plant height (>25%) and leaf number (ca. 20%), and decreased leaf area (Figures 3, 4; Supplemental Figures 1, 3, 4). Further, as indicated by the FW *versus* DW values of WT and AS-*SAMDC*, WT appeared to maintain a more favorable relative water content than AS-*SAMDC*. Unlike our previous data in which biomass of young seedlings overexpressing *Zea mays PAO* slightly decreased under control conditions (Moschou et al., 2008a), AS-*SAMDC* biomass of adult plants was not. This difference highlights that PA titers *per se* may not be that important for vigor of plants grown under normal conditions. A possible explanation for the increased height and leaf number of AS-*SAMDC* could be sought in their ability to grow faster compared to WT under normal conditions. As such, metabolism of transgenic AS-*SAMDC* plants seems to be more active and therefore more vulnerable to redox changes.

NaCl stress induces redox changes in the cells. Thus, NaCl treatment led to significant impairment of AS-*SAMDC* development. Among the traits measured, total leaf area was mostly affected, indicating that leaf expansion is very sensitive to NaCl treatments. In particular at mild NaCl stress (100 mM), which imitates better field stress conditions than any other NaCl concentration, WT had slightly increased total leaf area than AS-*SAMDC* (ca. 10%; Supplemental Figure 2). The latter showed increased sensitivity and a sharp decrease in biomass production (both on FW and DW basis), height and leaf number. Consistent with previous reports showing that plant height as a trait is not strictly associated with tolerance (Morales et al., 2012), AS-*SAMDC* did not show increased tolerance to NaCl stress. Taken together, our results allow suggesting a trade-off model in which NaCl tolerance and biomass production antagonize, with PAs exerting a possible regulatory role.

The increased biomass in AS-*SAMDC* under control conditions and at mild NaCl stress poses an interesting question: which is the molecular mechanism that underpins this increase? Deregulation of regulatory elements that confer increased tolerance to abiotic stress is usually accompanied by growth handicap and yield penalties due to cross-talk between developmental and stress-response networks (Cabello et al., 2014). For example, in intra-specific *Arabidopsis* hybrids, stress-responsive genes are repressed under normal conditions but are induced to mid-parent or higher levels under stress at certain times of the day, potentially buffering the trade-off between stress responses and growth (Miller et al., 2015). In the same study, repression of two stress-responsive genes increased growth vigor. We suggest that repression of metabolites such as PAs may also regulate stress/yield balances. Previously we showed that the PAs catabolic pathway is a regulator of the trade-off between abiotic and biotic stress (Moschou et al., 2009). This finding is particularly relevant for plants grown in natural environments that are often simultaneously challenged by a combination of stresses, which lead to synergistic, neutral, or even antagonistic effects. Herein, we extent our findings by showing that reduction of PAs leads to increased plant vigor under normal growth conditions if this is not accompanied by increased PA oxidation.

In *Arabidopsis* hybrids, increased vigor has been associated with enhanced photosynthesis (Fujimoto et al., 2012). We showed that maximal photosynthetic rate was highly variable and did not show a constant trend (Figure 5). However the actual photosynthesis under growth chamber/greenhouse conditions could be higher in AS-*SADMC* providing the underlying cause of higher biomass. For example the proton flux from lumen to stroma was higher in AS-*SAMDC* at light intensity similar to the one used under our growth conditions (ca. 40%; Figure 6). The remarkable decrease of A_*net*_ in both genotypes under NaCl stress was not due to decreased stomatal conductance, contrary to previous observations (Yang and Lu, 2005; Shahid et al., 2011).

The efficiency of photosynthesis (expressed by chlorophyll fluorescence or QY of PSII) under NaCl stress, has been evaluated in a number of species, with contradictory results so far. It is worth noticing that previous studies (for example Misra et al., 2001) showed that chlorophyll fluorescence was not a reliable indicator of NaCl tolerance. In the AS-*SAMDC* plants, low PA titers in the photosynthetic apparatus were accompanied by very low qE (Figure 6). The qE is one of the main molecular switches for the protection of the plant and the efficiency of photosynthetic machinery (Ruban et al., 2007). The results in Figure 6 provide genetic support of previous reports showing that elevation of PAs increases qE in tobacco (Ioannidis and Kotzabasis, 2007), in green algae (Ioannidis et al., 2011) and in isolated light harvesting complexes from tobacco (Tsiavos et al., 2012). Furthermore, qE is increased due to NaCl stress much more intensively in AS-*SAMDC* than in WT, although the absolute value of qE is still low (1.08 ± 0.1 for WT and 0.78 ± 0.11 for AS-*SAMDC*). Consistently, previous studies demonstrated that NaCl stress increases qE (Stepien and Johnson, 2009; Takizawa et al., 2009; Azzabi et al., 2013). The cause of qE increase in AS-*SAMDC* might be their increased susceptibility to NaCl stress.

During severe NaCl stress, photosynthesis would likely need to operate under conditions where the ionic strength inside the plastid is high. In this case, *pmf* storage would be heavily biased toward ΔpH formation (Robinson et al., 1983; Ioannidis et al., 2012a). For WT, ΔpH/*pmf* was about 0.52 (at 490 μmol photons m^−2^s^−1^) and increased to about 0.67 with NaCl. AS-*SAMDC* (control) traces were not adequately deconvoluted with the equations used for the WT and thus we could not estimate corresponding ΔpH/*pmf* values. In all cases, energy dissipation would be more easily and strongly induced at low and moderate light intensities, severely limiting the productivity and growth of the plant, even if water and CO_2_ are not limiting. Thus, the accumulation of PAs observed in plants grown under high NaCl stress (Alcazar et al., 2006) could serve as a way to increase the buffering of lumen, rebalancing *pmf* toward ΔΨ and optimizing the regulation of energy transduction. PAs could have a direct effect on this partitioning, *via* pH buffering (Ioannidis et al., 2012a) or by affecting the thylakoid ion conductance (Shabala and Pottosin, 2014; Pottosin and Shabala, 2016).

As a cautionary note we should state that estimation with high precision of chloroplastic PAs is refractory to our current analytical methods. Soluble PAs (and amines in general) diffuse rapidly (within seconds) outside chloroplast during isolation. The steady state uptake of low molecular weight amines (such as hexylamine) is reached within 15 s (Pick and McCarty, 1980) and it is accepted that the accumulation of amines has half times 2.5–4 s [Horner and Moudrianakis, 1986 and refs 16,17, and 20 therein]. PAs that are estimated in thylakoids correspond mainly to the fraction of PAs that is covalently bound to macromolecules by specific plastidic transglutaminases (Ioannidis et al., 2012b). The amount of thylakoid-associated PAs seems less sensitive to the large decrease evident at the leaf level. This stricter homoeostatic control indicates that they are probably essential for leaf physiology and photosynthetic performance. Early studies showed that chloroplast development (Dornemann et al., 1996), photoadaptation (Kotzabasis et al., 1999), and abiotic stress (Sfichi et al., 2004) in green algae, as well as de-etiolation in higher plants (Andreadakis and Kotzabasis, 1996), and abiotic stress in general (Lutz et al., 2005; Demetriou et al., 2007) is strongly associated with changes in the amount of thylakoid associated PAs and especially of Put.

ROS accumulation is a well-established pan-organismal hallmark of abiotic/biotic stresses. Despite their harmful nature at high concentrations, there is now compelling evidence that ROS are also powerful signaling molecules involved in the acclimation response to stress stimuli (Bailey-Serres and Mittler, 2006; Skopelitis et al., 2006). The significantly higher SOD activity, which dismutates O${}_{2}^{.-}$ to form H_2_O_2_, and the increased NADPH-oxidase suggests that SAMDC affects the regulation of NADPH-oxidase derived H_2_O_2_ (Figure 7). These rather moderate increases of antioxidants did not lead to vigor loss, as reported previously for *PAO* overexpressing plants (Moschou et al., 2008a). In addition, NADPH-oxidase has been linked to developmental processes including cell elongation (Sagi and Fluhr, 2006). Indeed, both NADPH-oxidase and SOD increased in both genotypes, while CAT remained fairly constant. Notably, mRNA levels of NADPH-oxidase and SOD, did not coincide with corresponding enzymatic activities (under control and NaCl stress conditions), suggesting that these enzymes are controlled at the post-translational level. Whether the increased NADPH-oxidase activity in AS-*SAMDC* drives the increased biomass in these plants remains to be shown.

NaCl stress led to H_2_O_2_ accumulation in WT (Figure 8). The AS-*SAMDC* control plants accumulated marginally higher H_2_O_2_ content, with NaCl stress inducing a large increase of H_2_O_2_ production in this genotype. Furthermore, although AS-*SAMDC* treated for 24 h with 100 mM showed a significant increase of total phenolics (Figure 9) they could not restore H_2_O_2_ to WT levels. Changes in phenolic content have been associated with biotic and abiotic stresses, such as wounding, drought, NaCl, metal toxicity and nutrient deprivation (Parida and Das, 2005). Herein, higher content of soluble phenolic compounds of AS-*SAMDC versus* WT was recorded over a period of 21 DAT (Supplemental Figure 9A). Although plants with increased phenolic content are considered more tolerant to stresses, our findings do not support a better performance of AS-*SAMDC* at least against NaCl.

PAs are major regulators of ion channel conductance. Ion uptake is crucial not only for normal growth but also for growth under NaCl stress (Parida and Das, 2005); limitations in plant growth may, to some extent, be due to disruption of ion homeostasis. NaCl tolerance is usually associated with the ability of cells to restrict, exclude, compartmentalize, or transport toxic ions from roots to shoots (Attia et al., 2011). In the soil, high concentrations of soluble salts, such as Ca^2+^, Mg^2+^, and Na^+^ from cations, contribute to the high electrical conductivity of saline soils (Chinnusamy et al., 2006). A systematic quantitative analysis of K^+^ and rubidium levels on several *Arabidopsis* natural accessions showed that polyploid accessions exhibit higher K^+^ content in leaves, characteristic that correlates with NaCl tolerance (Chao et al., 2013). Under control conditions, AS-*SAMDC* and WT showed similar ion concentrations. Total major ions content is mainly defined by vacuoles (Pottosin and Shabala, 2014). The only Ca^2+^ and Mg^2+^-permeable channel, characterized in the vacuolar membrane so far, is the tonoplast slow vacuolar (TPC1/SV) channel (Bonales-Alatorre et al., 2013). It was shown that TPC1/SV relative expression controls the re-distribution of Ca^2+^ between epidermis and mesophyll vacuoles: the higher the TPC1 expression, the lower the Ca^2+^ accumulation (Gilliham et al., 2011). PAs are efficient natural blockers of the SV channel; their decrease is expected to increase the leak of Ca^2+^ and Mg^2+^ (and also K^+^) *via* SV channels and will tend to decrease the accumulation of these ions in the vacuoles (hence, in the whole tissue). At 0 DAT we did not observe significant differences between genotypes regarding ion accumulation (Supplemental Table 3). However, as time progressed AS-*SAMDC* showed reduced accumulation of Na^+^, Ca^2+^ and Mg^2+^ under control conditions (Figure 10; 7-21 DAT). At severe stress (300 mM NaCl) the observed damage was greater in the transgenic AS-*SAMDC* plants, probably due to the higher accumulation of Na^+^ in the shoot. It has been previously shown that when tolerant plants were grown under NaCl conditions, they maintained higher K^+^ and lower Na^+^ content in the cytoplasm, by regulating the expression of ion transporters (Tester and Davenport, 2003). At lower NaCl concentrations however, no significant differences were observed between the WT and AS-*SAMDC* in the accumulation of Na^+^.

Potassium content in both genotypes increased in parallel to the severity of stress, and was significantly higher only at 200 and 300 mM NaCl with AS-*SAMDC* accumulating constantly lower levels of K^+^ in the shoots than the WT. Increased K^+^ content in shoots of plants grown under salt stress has been previously reported (Hariadi et al., 2011; Adem et al., 2014), and it has been often considered as a tolerance trait. As far as Ca^2+^ is concerned, its increase during abiotic and biotic stresses is required for tolerance in *Arabidopsis* (Johnson et al., 2014). Previously, we showed that over-accumulation of Ca^2+^ in pollen tubes induced cell death (Wu et al., 2010). In plant cells the resting cytoplasmic content of Ca^2+^, [Ca^2+^]cyt, under normal conditions is maintained in the range of 50–200 nM, whereas the content of Ca^2+^ in cell wall, vacuole, endoplasmic reticulum, and mitochondria is 1–10 mM. However, specific signals, such as NaCl stress can trigger a sudden increase in the [Ca^2+^]cyt to mM level that is toxic if it persists for longer time in the cytoplasm (Kader and Lindberg, 2010). Therefore, plants have evolved a system to take up excess Ca^2+^ and store it either in the apoplast or into the lumen of intercellular organelles, such as vacuole or endoplasmic reticulum. The latter, together with cell walls, can be used for elevating the [Ca^2+^]cyt level defining the appropriate signatures under stress conditions to transduce the signal to subsequent downstream defense responses. Interestingly, a similar accumulation pattern was observed for Mg^2+^ in AS-*SAMDC* suggesting that divalent cations may be controlled in a similar manner by PAs during NaCl. In addition, low PAs content in AS-*SAMDC* during NaCl stress does not seem to affect loss of ions from the vacuoles, suggesting that another channel/pathway might be involved in preventing ion leakage.

Taken together, our results suggest that SAMDC is a putative modulator of the trade-off between stress tolerance and plant growth and developmental traits. Unlike previous reports, we show that SAMDC depletion leads to increased plant vigor under normal growth conditions by affecting photosynthesis, ROS homeostasis and ion content. The reported positive effect of SAMDC depletion is nullified during abiotic stress, and instead AS-*SAMDC* plants show increased sensitivity. These results can be used as the basis for designing strategies to increase tolerance to NaCl omitting associated yield penalties.

However, it is well known that salinity stress tolerance is a polygenic trait and many processes are regulated by PAs homeostasis. The results herein reveal the behavior of AS-*SAMDC* transgenics compared to WT regarding certain growth and developmental characteristics, as well as molecular/biochemical and biophysical responses under NaCl stress, without differentiating between osmotic and ionic stress. To this aim, more work is required to clarify this issue.

## Author contributions

KR, KK, HC designed research; IM, CV, NI, CP, KG, EA, AR, DB, KH, AK, TM performed research; IM, PM, HC, KR wrote the manuscript.

### Conflict of interest statement

The authors declare that the research was conducted in the absence of any commercial or financial relationships that could be construed as a potential conflict of interest.
