# Supplementary material for: Silencing S-Adenosyl-L-Methionine Decarboxylase (SAMDC) in Nicotiana tabacum Points at a Polyamine-Dependent Trade-Off between Growth and Tolerance Responses
Source: Front Plant Sci. 2016 Mar 31;7:379. doi: 10.3389/fpls.2016.00379 (PMC4814703; doi:10.3389/fpls.2016.00379)
Supplement: Supplementary file 1 [file DataSheet1.DOC]

Supplemental Material

***Nicotiana tabacum* Plants with Silenced S-Adenosyl-L-Methionine Decarboxylase (*SAMDC*) Points at a Polyamine-dependent Trade-off between Growth and Tolerance responses**

**Ifigeneia Mellidou^1^, Panagiotis N. Moschou^2^, Nikolaos E. Ioannidis^4^, Chryssa Pankou^1^, Katalin Gėmes^5^, Chryssanthi Valassakis^3^, Efthimios A. Andronis^4^, Despoina Beris^3^, Kosmas Haralampidis^3^, Andreas Roussis^3^, Aikaterini Karamanoli^1^, Theodora Matsi^1^, Kiriakos Kotzabasis^4^, Helen-Isis Constantinidou^1*^, Kalliopi A. Roubelakis-Angelakis^4*^**

*** Correspondence:**

Kalliopi A. Roubelakis-Angelakis, poproube@biol.uoc.gr

Helen-Isis Constantinidou, constad@agro.auth.gr

**Supplemental Information**

**The salt stress is the sum of salinity and sodicity under our conditions**

It is well known that the characteristics of the soil substrate significantly affect the water/soluble constituents’ uptake by plants. The pH of the culture medium (substrate) was acidic, typical of peat, and in all cases ranged from 5.5 to 6.5. However from the 7 DAT and afterwards, EC_se_ and SAR of the substrate increased significantly (*P*≤0.001) with increasing NaCl application rates. At all rates, both EC_se_and SAR attained high values (Supplemental Table 1), as it was evident from the (a) EC_se_of the saturation extract, which was higher than the critical limit of 4 dS m^-1^recorded for normal growth in most crops and (b) SAR which was higher than the critical value of 13(Brady and Weil, 2008). Thus, the adverse effects observed on the different plant parameters were the result of both salinity as well sodicity stress (‘NaCl stress’). The ANOVA indicated that all the parameters examined in this study were significantly (*P*≤0.001) affected (Table 1) by sampling time and by the five different NaCl concentrations. In most cases, the effect of the genotype, as well as the effect of the two-way interactions on the various parameters were also significant (Supplemental Table 1). As we were particularly interested in evaluating the effects of NaCl stress on transgenic tobacco downregulating the *SAMDC* gene and in order to facilitate comparisons, means across the four sampling points were averaged per salt concentration over the entire experimental period (up to 21 DAT).Moreover, as a result of NaCl addition, water soluble Na^+^ of the substrates increased following the NaCl application rates. However, an increasing trend of the other water soluble cations was also evidenced with increasing NaCl rates (Supplemental Table 1). This increase was attributed to the substitution of the substrates' exchangeable cations by the added Na^+^.

**Supplemental Table 1.** Mean values of EC, SAR and water soluble cations of the substrates.The standard deviation is reported in parentheses.

| NaCl | EC | SAR | Na | K | Ca | Mg |
| --- | --- | --- | --- | --- | --- | --- |
| (mM) | dS m^-1^ |  | mmol_c_ L^-1^ | | | |
| 0 | 1.1 (0.3) | 3.2 (0.7) | 5.7 (1.6) | 0.30 (0.16) | 4.9 (2.6) | 1.8 (0.8) |
| 50 | 6.9 (1.3) | 17.0 (3.0) | 49.4 (10.2) | 0.24 (0.08) | 13.0 (2.4) | 3.9 (0.9) |
| 100 | 11.2 (2.5) | 22.8 (3.7) | 78.0 (15.6) | 0.30 (0.08) | 18.2 (3.2) | 5.2 (0.9) |
| 200 | 18.8 (2.2) | 42.2 (6.0) | 169 (20) | 0.50 (0.17) | 26.2 (5.1) | 6.5 (0.8) |
| 300 | 24.8 (4.3) | 54.9 (9.3) | 237 (44) | 0.62 (0.13) | 30.1 (5.2) | 7.3 (1.1) |

The ANOVA indicated that all the parameters examined in this study were significantly (*P*≤ 0.001) affected (Supplemental Table 2) by sampling time and by the five different NaCl concentrations. In most cases, the effect of the genotype, as well as the effect of the two-way interactions on the various parameters were also significant (Supplemental Table 2).

**Supplemental Table 2.**Results of analysis of variance (ANOVA) applied on attributes of the genotypes examined in the presence of five concentrations of NaCl (0, 50, 100, 200, 300 mM). Given is the F-ratios’ significance for the effects exerted by the genotype (G), the salt concentration (S), and the sampling time (T). The different parameters were determined using 70-day old tobacco grown in pots in a greenhouse. Asterisks indicate significant differences at *P*≤0.05 (*), at *P*≤0.01(**), and at *P*≤0.001 (***). NS; non-significant.

|  | Significance of F-ratio | | | | | | | | | | | | | |  | |
| --- | --- | --- | --- | --- | --- | --- | --- | --- | --- | --- | --- | --- | --- | --- | --- | --- |
| Variation Source | df | Plant height | Leaf area | Number of leaves | Fresh weight | Dry weight | Na^+^ | K^+^ | Ca^++^ | Mg^++^ | A_net_ | QY^a^ | CCI^b^ | Total soluble phenolic compounds | | Antioxidant capacity(FRAP) |
| G | 1 | ** | * | ** | * | ** | * | ** | * | * | NS | ** | NS | * | | * |
| S | 4 | *** | *** | *** | *** | *** | *** | *** | *** | *** | *** | *** | *** | *** | | *** |
| G x S | 4 | * | NS | * | NS | *** | * | * | *** | ** | NS | * | NS | NS | | NS |
| T | 3 | *** | ** | *** | *** | *** | *** | *** | *** | *** | *** | *** | *** | *** | | *** |
| T x G | 3 | ** | NS | ** | * | * | ** | * | *** | *** | NS | ** | ** | * | | * |
| T x S | 12 | *** | *** | NS | *** | *** | *** | *** | *** | *** | *** | *** | *** | *** | | *** |
| T x G x S | 12 | NS | NS | NS | NS | NS | ** | NS | * | ** | * | NS | NS | NS | | NS |

^a^ Quantum Yield of PSII

^b^ Chlorophyll Content Index

**Supplemental Table 3**. Ion contents in 70-day old WT and AS*-SAMDC* tobacco plants at 0 DAT. Each mean represents the average of three replications; NS, non-significant at *P*<0.05.

|  | **WT** | | **AS-*SAMDC*** | |  |
| --- | --- | --- | --- | --- | --- |
|  | **mean** | **SD** | **mean** | **SD** | **Level of significance** |
| Na^+^ content (g kg^-1^ DW) | 1.88 | 0.26 | 1.76 | 0.17 | NS |
| K^+^ content (g kg^-1^ DW) | 31.4 | 6.17 | 30.2 | 8.47 | NS |
| Ca^++^ content (g kg^-1^ DW) | 7.67 | 2.73 | 6.37 | 1.34 | NS |
| Mg^++^ content (g kg^-1^ DW) | 2.78 | 0.75 | 2.83 | 0.32 | NS |

**REFERENCES**

Brady, N., and RR Weil (2008). The nature and properties of soils. 14th edition. Pearson Prentice Hall, Upper Saddle River, NJ

**Supplemental Table 4.** Absolute values of NPQ used in Figure 6.

| **NPQ** |  | **WT** | **SE** | **WT (200mM)** | **SE** | **AS-SAMDC** | **SE** | **AS-SAMDC (200 mM)** | **SE** |
| --- | --- | --- | --- | --- | --- | --- | --- | --- | --- |
| **64** |  | 0,162 | 0,0035 | 0,206 | 0,016 | 0,166 | 0,01409 | 0,222 | 0,0136 |
| **181** |  | 0,510 | 0,0057 | 0,716 | 0,058 | 0,316 | 0,05424 | 0,603 | 0,0367 |
| **366** |  | 1,100 | 0,0211 | 1,210 | 0,073 | 0,592 | 0,14793 | 0,988 | 0,0628 |
| **490** |  | 1,287 | 0,1449 | 1,345 | 0,071 | 0,829 | 0,17986 | 1,137 | 0,0624 |
| **qE** |  | **WT** | **SE** | **WT (200mM)** | **SE** | **AS-SAMDC** | **SE** | **AS-SAMDC (200 mM)** | **SE** |
| **64** |  | 0,122 | 0,0124 | 0,119 | 0,006 | 0,093 | 0,01663 | 0,105 | 0,0077 |
| **181** |  | 0,474 | 0,0303 | 0,604 | 0,077 | 0,218 | 0,02594 | 0,452 | 0,0686 |
| **366** |  | 1,020 | 0,0642 | 1,034 | 0,106 | 0,398 | 0,1107 | 0,738 | 0,1064 |
| **490** |  | 1,127 | 0,1948 | 1,084 | 0,099 | 0,474 | 0,1193 | 0,787 | 0,1079 |
| **ECSt** |  | **WT** | **SE** | **WT (200mM)** | **SE** | **AS-SAMDC** | **SE** | **AS-SAMDC (200 mM)** | **SE** |
| **64** |  | 0,00093 | 0,0001 | 9,34E-04 | 0,00002 | 0,0009 | 5,37E-05 | 9,58E-04 | 0,00001 |
| **181** |  | 0,00264 | 0,0004 | 0,00249 | 0,00005 | 0,0020 | 1,68E-04 | 0,00258 | 0,00015 |
| **366** |  | 0,00318 | 0,0001 | 0,0034 | 0,00004 | 0,0029 | 2,05E-04 | 0,00336 | 0,00022 |
| **490** |  | 0,00337 | 0,0002 | 0,00358 | 0,00007 | 0,0031 | 2,61E-04 | 0,00343 | 0,00022 |
| **LEF** |  | **WT** | **SE** | **WT (200mM)** | **SE** | **AS-SAMDC** | **SE** | **AS-SAMDC (200 mM)** | **SE** |
| **64** |  | 35,52 | 1,9303 | 36,75 | 0,307 | 33,52 | 0,22167 | 33,61 | 0,07 |
| **181** |  | 76,27 | 5,7563 | 73,47 | 1,067 | 72,63 | 3,2387 | 65,69 | 0,45 |
| **366** |  | 107,48 | 13,3170 | 83,62 | 1,688 | 94,86 | 9,46539 | 74,25 | 1,08 |
| **490** |  | 108,41 | 15,8337 | 85,17 | 3,559 | 95,74 | 12,5514 | 72,70 | 0,49 |
| **gH+** |  | **WT** | **SE** | **WT (200mM)** | **SE** | **AS-SAMDC** | **SE** | **AS-SAMDC (200 mM)** | **SE** |
| **64** |  | 29,19 | 0,9715 | 29,58 | 2,56 | 42,39 | 11,46727 | 34,41 | 1,26 |
| **181** |  | 49,81 | 4,1531 | 44,36 | 0,73 | 63,82 | 0,94865 | 47,13 | 2,21 |
| **366** |  | 57,28 | 6,6005 | 44,28 | 0,91 | 65,47 | 0,82333 | 48,52 | 2,52 |
| **490** |  | 58,39 | 6,9486 | 45,13 | 0,53 | 63,89 | 0,78413 | 51,48 | 2,44 |
| **UH+** |  | **WT** | **SE** | **WT (200mM)** | **SE** | **AS-SAMDC** | **SE** | **AS-SAMDC (200 mM)** | **SE** |
| **64** |  | 0,027 | 0,0007 | 0,028 | 0,0027 | 0,039 | 0,01299 | 0,033 | 0,0010 |
| **181** |  | 0,131 | 0,0207 | 0,110 | 0,0004 | 0,125 | 0,0126 | 0,121 | 0,0028 |
| **366** |  | 0,183 | 0,0249 | 0,151 | 0,0039 | 0,188 | 0,0151 | 0,162 | 0,0068 |
| **490** |  | 0,199 | 0,0323 | 0,161 | 0,0030 | 0,200 | 0,0183 | 0,175 | 0,0067 |


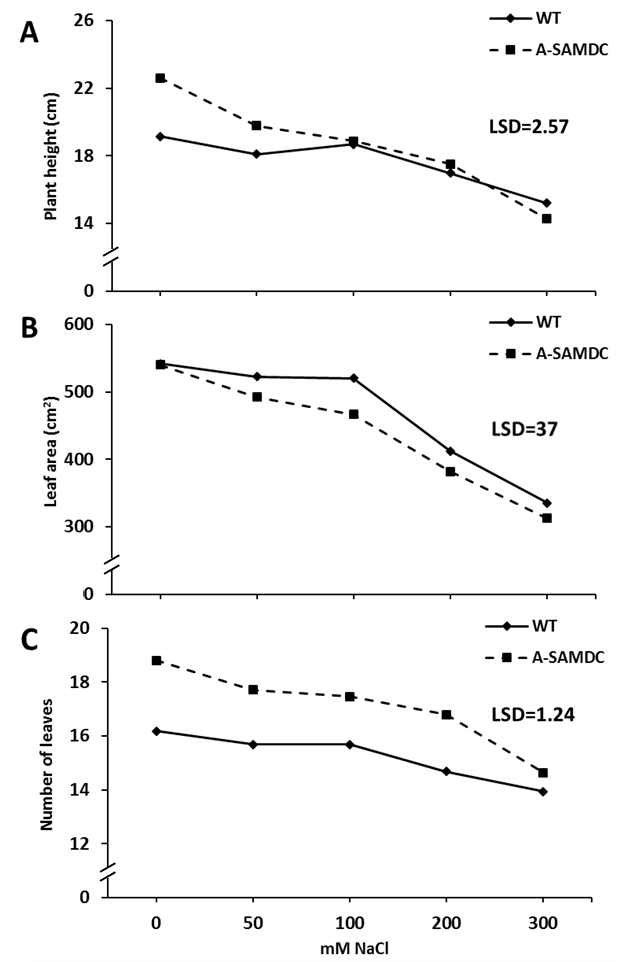


**Supplemental Figure 1.** (Α) Plant height (cm), (Β) leaf area (cm^2^), and (C) number of leaves per plant of tobacco WT and *AS-SAMDC* plants exposed to different salt levels (0, 50, 100, 200, 300 mM). Means are averaged over the whole experimental period.

**
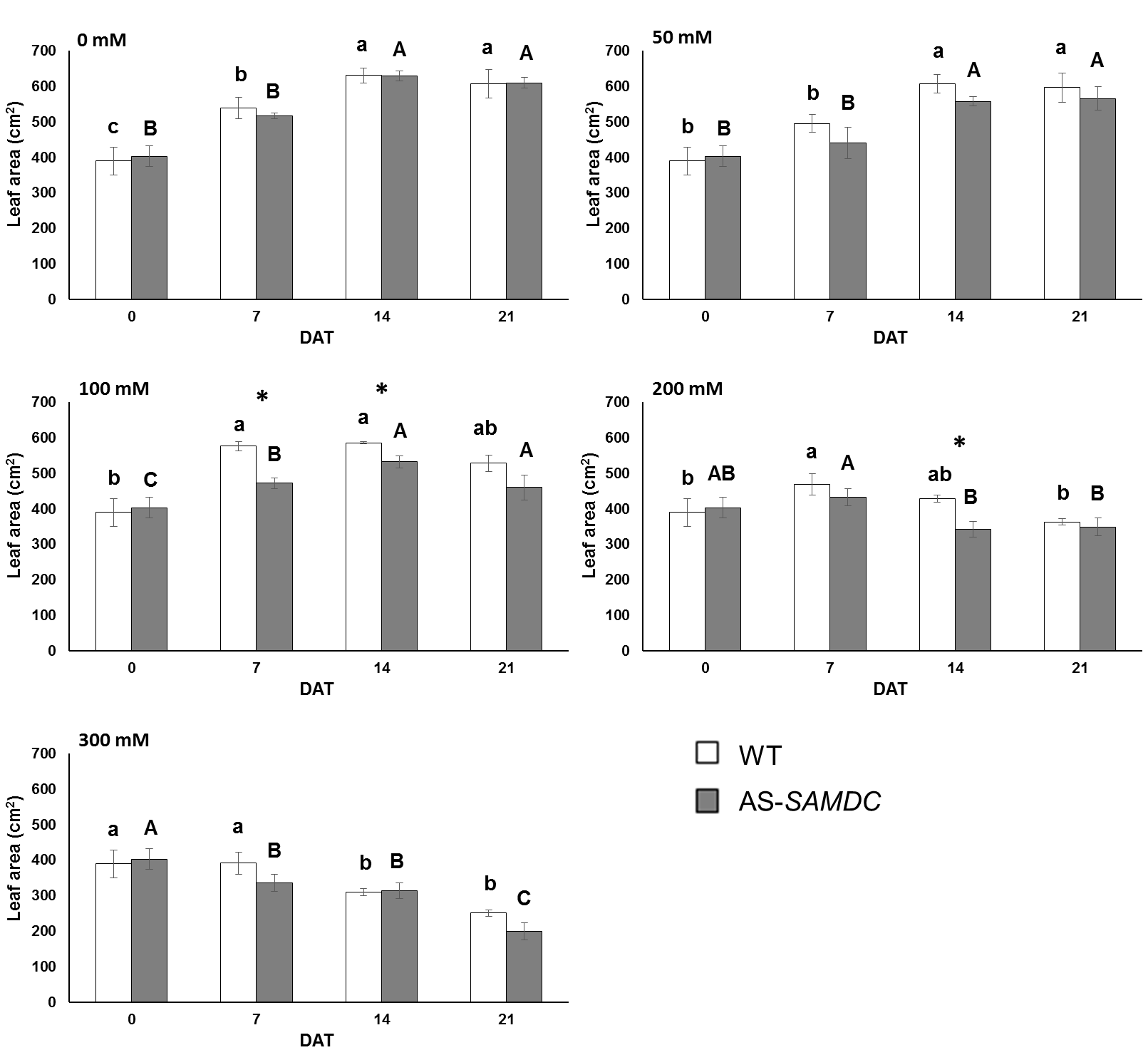
**

**Supplemental Figure 2.**Leaf area (cm^2^) of WT and AS-*SAMDC* exposed to different NaCl concentrations (0, 50, 100, 200, 300 mM), 0, 7, 14 and 21 DAT. Data are means ±SE. Different letters (lower case for WT, upper case for AS-*SAMDC*) indicate significant differences over time within the same genotype, based on Tukey's HSD test (*P*<0.05). Asterisks indicate significant difference of mean values of AS-*SAMDC* from WT (Student’s t-test; * *P*<0.05, ***P*<0.01 and ****P*<0.001).

**
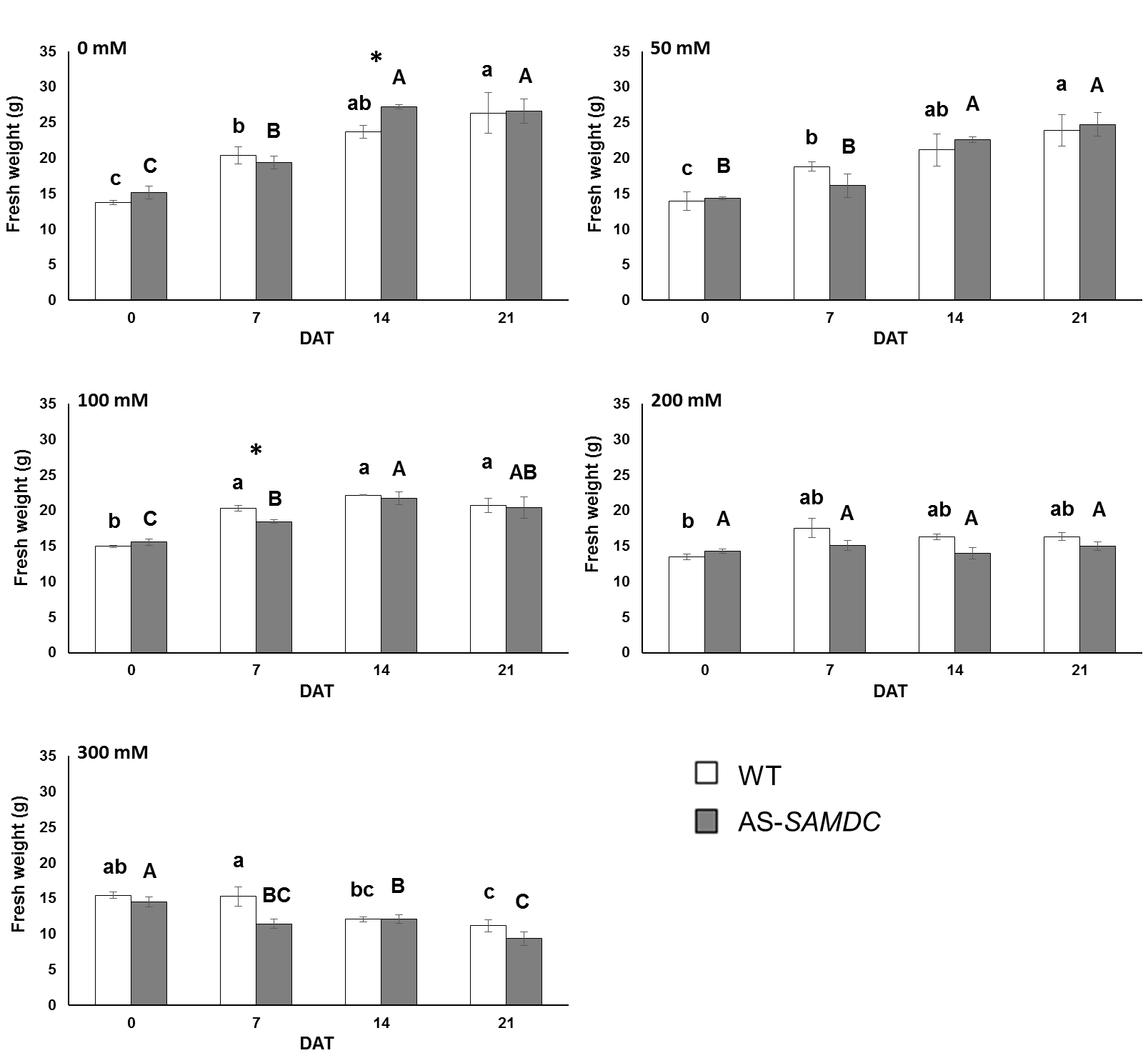
**

**Supplemental Figure 3.** Fresh weight (g) of WT and AS-*SAMDC* exposed to different NaCl concentrations (0, 50, 100, 200, 300 mM), 0, 7, 14 and 21 DAT. Data are means ±SE. Different letters (lower case for WT, upper case for AS-*SAMDC*) indicate significant differences over time within the same genotype, based on Tukey's HSD test (*P*<0.05). Asterisks indicate significant difference of mean values of AS-*SAMDC* from WT (Student’s *t*-test; * *P*<0.05, ***P*<0.01 and ****P*<0.001).

**
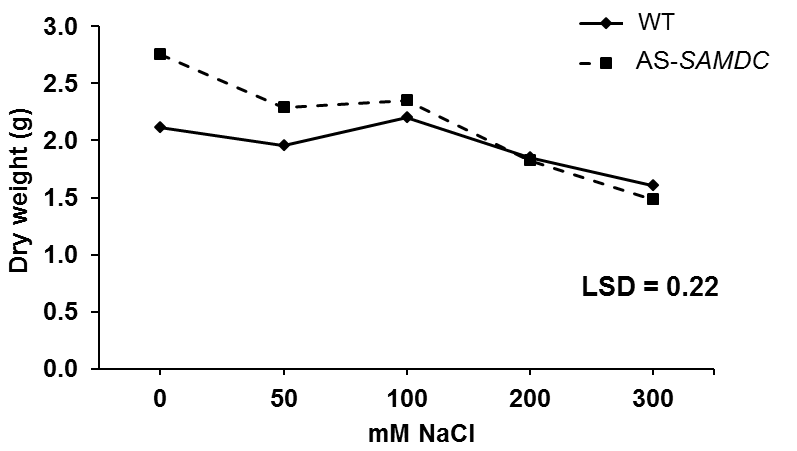
**

**Supplemental Figure 4.** Dry weight (g) of the above ground biomass of tobacco WT and *AS-SAMDC* exposed to different NaCl concentrations (0, 50, 100, 200, 300 mM). Means are averaged over the whole experimental period.

**
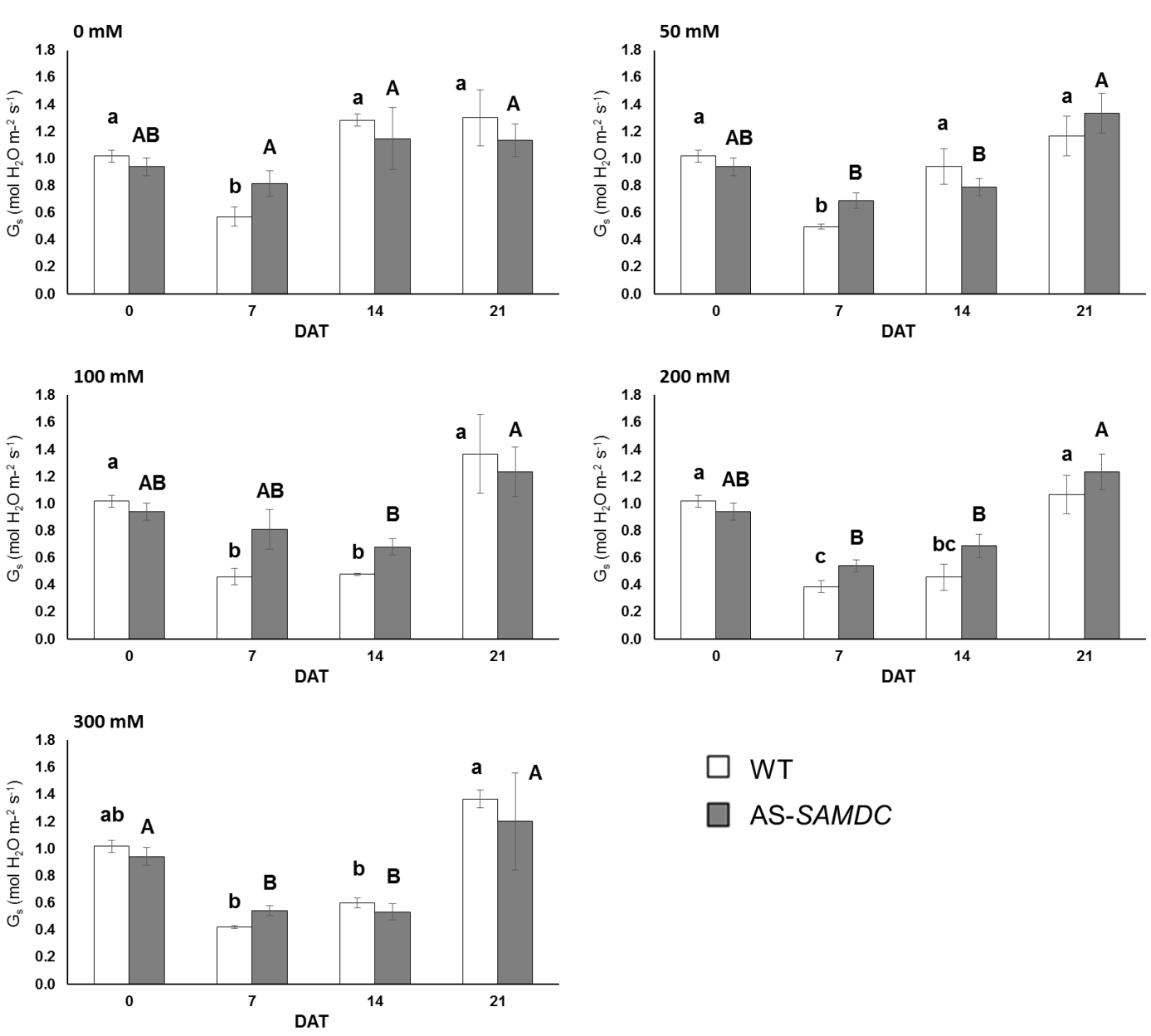
**

**Supplemental Figure 5.** Stomatal conductance (mol H_2_O m^-2^ s^-1^) of leaves of WT and AS-*SAMDC*exposed to different NaCl concentrations (0, 50, 100, 200, 300 mM), 0, 7, 14 and 21 DAT. Data are means ±SE. Different letters (lower case for WT, upper case for AS-*SAMDC*) indicate significant differences over time within the same genotype, based on Tukey's HSD test (*P*<0.05). Asterisks indicate significant difference of mean values of AS-*SAMDC* from WT (Student’s *t*-test; * *P*<0.05, ***P*<0.01 and ****P*<0.001).


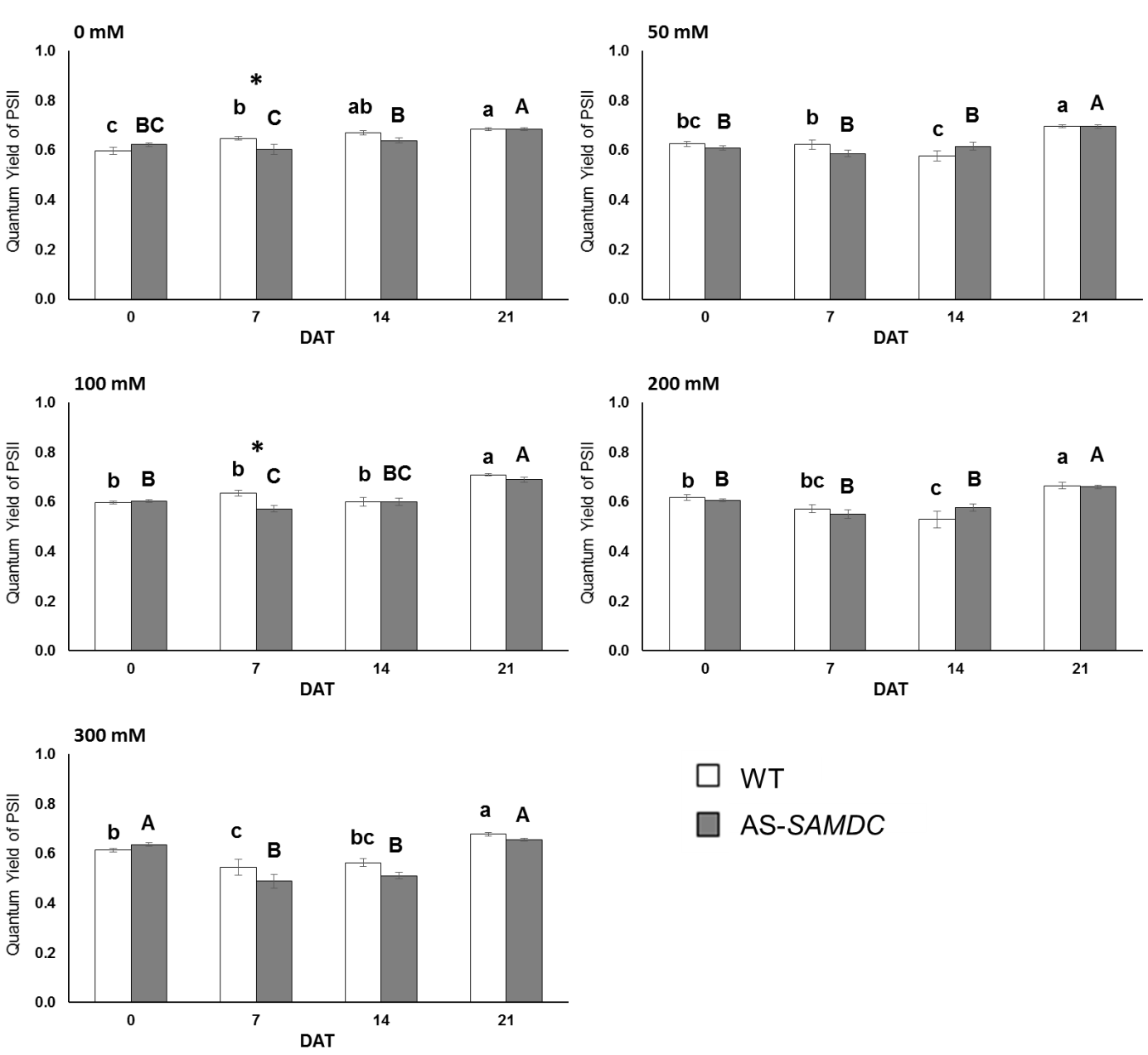


**Supplemental Figure 6.** Quantum yield PSII of leaves of WT and AS-*SAMDC* exposed to different NaCl concentrations (0, 50, 100, 200, 300 mM), 0, 7, 14 and 21 DAT. Data are means ±SE. Different letters (lower case for WT, upper case for AS-*SAMDC*) indicate significant differences over time within the same genotype, based on Tukey's HSD test (*P*<0.05). Asterisks indicate significant difference of mean values of AS-*SAMDC* from WT (Student’s t-test; * *P*<0.05, ***P*<0.01 and ****P*<0.001).

**
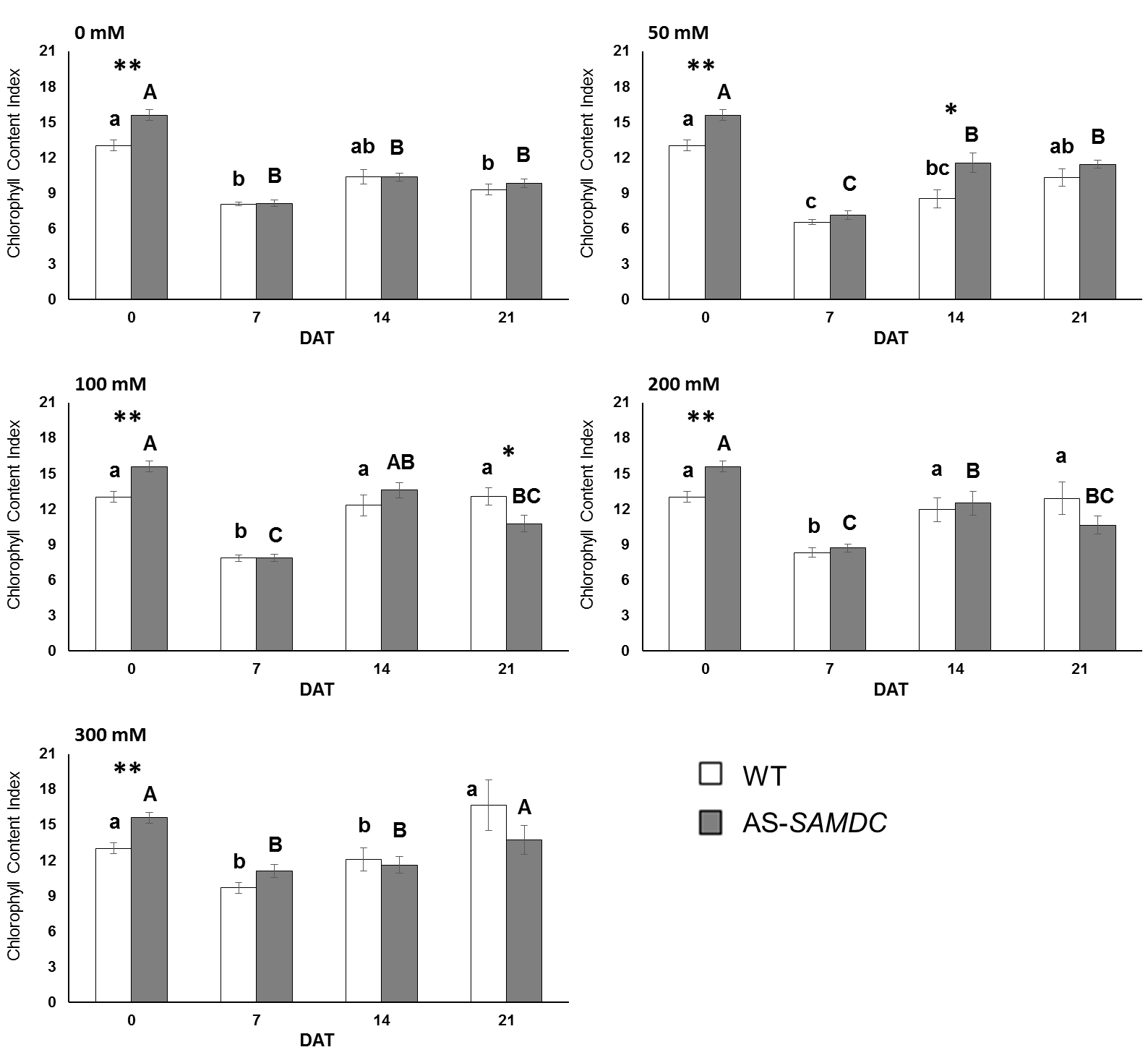
**

**Supplemental Figure 7.** Chlorophyll content index of leaves of WT and AS-*SAMDC* exposed to different NaCl concentrations (0, 50, 100, 200, 300 mM), 0, 7, 14 and 21 DAT. Data are means ±SE. Different letters (lower case for WT, upper case for AS-*SAMDC*) indicate significant differences over time within the same genotype, based on Tukey's HSD test (*P*<0.05). Asterisks indicate significant difference of mean values of AS-*SAMDC* from WT (Student’s *t*-test; * *P*<0.05, ***P*<0.01 and ****P*<0.001).

**
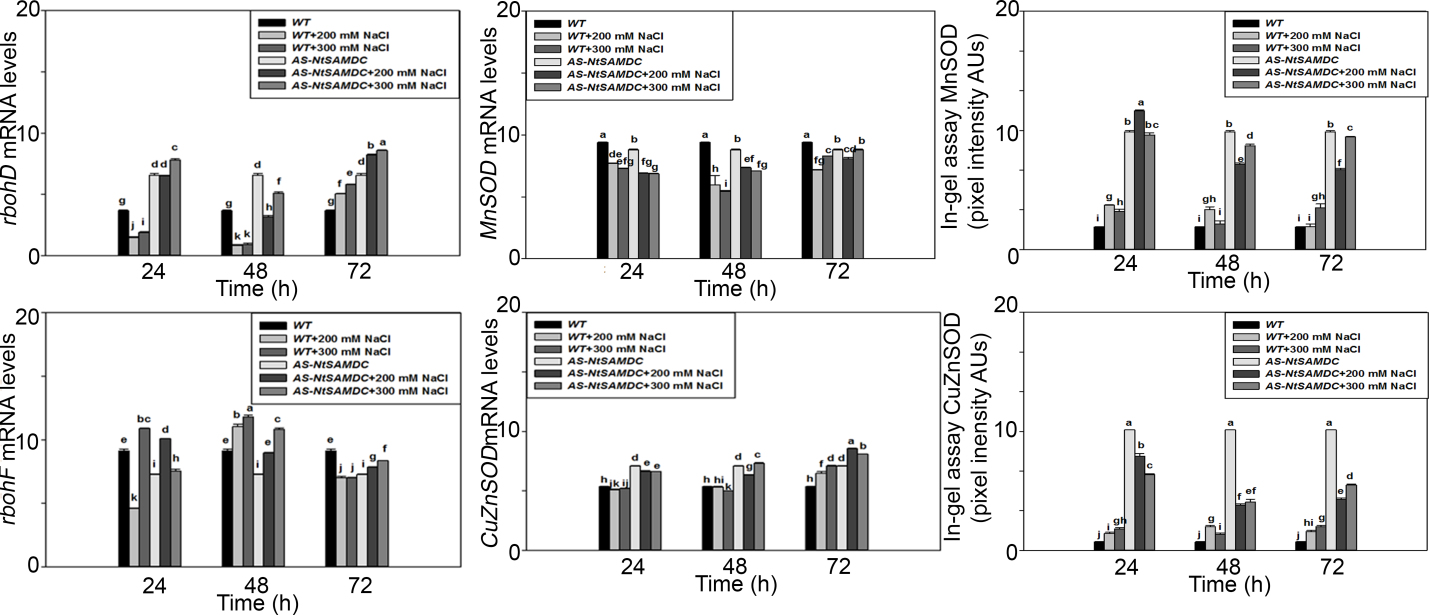
**

**Supplemental Figure 8.** mRNA levels of *rbohD*, *rbohF*, *MnSOD*, *CuZnSOD* and in-gel activity of MnSOD and CuZnSOD. AUs, Arbitrary units. Data are means±SE of three biological replicates with three technical replicates each. Different letters indicate significant differences of Duncan’s multiple comparisons (*P*<0.05).

**
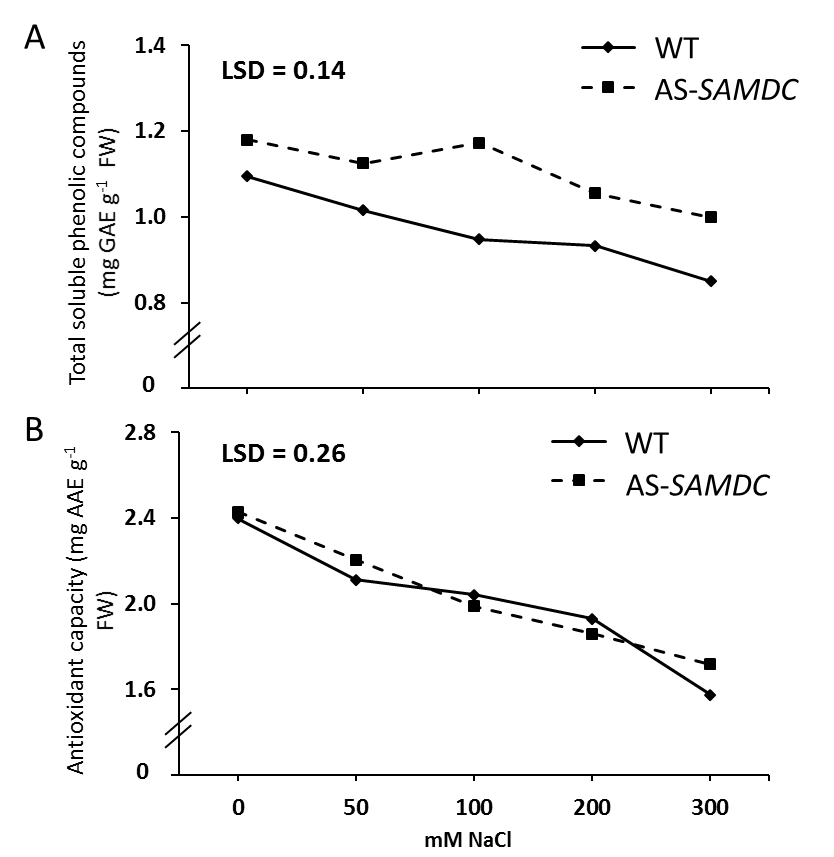
**

**Supplemental Figure 9.** Total soluble phenolic compounds (mg GAE g^-1^ FW; A), and antioxidant capacity (mg AAE g^-1^ FW; B) of tobacco WT and AS-*SAMDC* exposed to different NaCl concentrations(0, 50, 100, 200, 300mM). Means are averaged over the whole experimental period.
